# Ancestral QTL Alleles from Wild Emmer Wheat Improve Drought Resistance and Productivity in Modern Wheat Cultivars

**DOI:** 10.3389/fpls.2016.00452

**Published:** 2016-04-15

**Authors:** Lianne Merchuk-Ovnat, Vered Barak, Tzion Fahima, Frank Ordon, Gabriel A. Lidzbarsky, Tamar Krugman, Yehoshua Saranga

**Affiliations:** ^1^The Robert H. Smith Institute of Plant Sciences and Genetics in Agriculture, The Hebrew University of JerusalemRehovot, Israel; ^2^Institute of Evolution and Department of Evolutionary and Environmental Biology, University of HaifaHaifa, Israel; ^3^Federal Research Centre for Cultivated Plants, Julius Kuehn-Institute, Institute for Resistance Research and Stress ToleranceQuedlinburg, Germany

**Keywords:** interspecific introgression, marker-assisted selection, near-isogenic line, *Triticum turgidum* ssp. *dicoccoides*, quantitative trait locus, water stress, wheat, yield

## Abstract

Wild emmer wheat (*Triticum turgidum* ssp. *dicoccoides*) is considered a promising source for improving stress resistances in domesticated wheat. Here we explored the potential of selected quantitative trait loci (QTLs) from wild emmer wheat, introgressed via marker-assisted selection, to enhance drought resistance in elite durum (*T. turgidum* ssp. *durum*) and bread (*T. aestivum*) wheat cultivars. The resultant near-isogenic lines (BC_3_F_3_ and BC_3_F_4_) were genotyped using SNP array to confirm the introgressed genomic regions and evaluated in two consecutive years under well-watered (690–710 mm) and water-limited (290–320 mm) conditions. Three of the introgressed QTLs were successfully validated, two in the background of durum wheat cv. Uzan (on chromosomes 1BL and 2BS), and one in the background of bread wheat cvs. Bar Nir and Zahir (chromosome 7AS). In most cases, the QTL x environment interaction was validated in terms of improved grain yield and biomass—specifically under drought (7AS QTL in cv. Bar Nir background), under both treatments (2BS QTL), and a greater stability across treatments (1BL QTL). The results provide a first demonstration that introgression of wild emmer QTL alleles can enhance productivity and yield stability across environments in domesticated wheat, thereby enriching the modern gene pool with essential diversity for the improvement of drought resistance.

## Introduction

Wheat (*Triticum* spp.) is one of the world's major food sources, providing about 20% of the calories consumed by mankind (FAO, 2011). The germplasm of semi-dwarf spring habit bread wheat, distributed by the International Maize and Wheat Improvement Center (CIMMYT) from the early 1960s to late 1980s along with advanced agronomic practices, led to substantial and rapid advances in yield (Fischer et al., 1998). This genetic improvement was mainly associated with an increased harvest index (HI) and a shift toward photoperiod-insensitivity rather than increased total biomass (Sayre et al., 1997; Ortiz et al., 2008). However, recent reviews suggest that the rate of yield progress in spring wheat has been slowing down for the last three decades (Fischer and Edmeades, 2010; Challinor et al., 2014; Curtis and Halford, 2014).

Drought, the major stress factor limiting crop productivity worldwide (Boyer, 1982; Araus et al., 2008), is expected to increase due to global climate change (Wheeler and Von Braun, 2013). Developing crop cultivars with improved drought resistance is considered a sustainable and an economically viable approach to enhance crop productivity and ensure food security for the growing human population. Past efforts to develop drought-resistant crop cultivars by traditional breeding were hampered by low heritability of traits such as yield, particularly under drought, and by large “genotype × environment” interactions (Blum, 1988; Passioura, 2012; Langridge and Reynolds, 2015). However, recent advances in molecular and genomic tools have enabled the identification of quantitative trait loci (QTLs) and diagnostic DNA markers in a wide range of crops, with the promise of accelerating crop improvement toward future challenges (Salvi and Tuberosa, 2015).

The available genetic diversity in crop plants has been considerably eroded compared with their wild ancestors, due to bottlenecks imposed by plant domestication (e.g., founder effect) and modern breeding (Tanksley and McCouch, 1997; Ladizinsky, 1998), thus making current crop germplasm vulnerable to various biotic and abiotic stresses. Therefore, a major objective of modern breeding is to enrich the domesticated gene pool by reintroducing valuable wild alleles that were “left behind” (Aaronsohn, 1910; Tanksley and McCouch, 1997; Gur and Zamir, 2004).

Wild emmer wheat, *Triticum turgidum* ssp. *dicoccoides* [Körn.] Thell., is the tetraploid (2n = 4x = 28; genome BBAA) progenitor of the domesticated tetraploid (2n = 4x = 28; BBAA) durum wheat [*T. turgidum* ssp. *durum* (Desf.) MacKey] and hexaploid (2n = 6x = 42; BBAADD) bread wheat (*T. aestivum* L.; Feldman, 2001). Wild emmer wheat evolved in the Near Eastern Fertile Crescent under a wide range of ecogeographical conditions (Peleg et al., 2005, 2008a) and harbors rich allelic diversity for numerous important traits, including agronomic characteristics, grain quality and resistance to biotic and abiotic stresses (Peng et al., 2012 and references therein). A large number of genes and QTLs that are valuable for wheat improvement have been identified in the wild emmer gene pool and mapped (Xie and Nevo, 2008). However, this gene pool has not been widely exploited in wheat breeding (Jaradat, 2011), possibly due to the complexity and long duration of gene introgression from wild germplasm. Marker-assisted selection has been shown effective for the introgression of favorable genes/QTLs, conferring primarily disease resistances (reviewed by Peng et al., 2012) from wild emmer wheat to domesticated germplasm. Marker assisted selection has also been used to transfer genes/QTLs conferring several agronomic traits to the domesticated gene pool, including Na^+^ exclusion (Munns et al., 2012), plant height (Lanning et al., 2012), tillering (Moeller et al., 2014), spike branching (Zhang et al., 2012), epicuticular wax (Miura et al., 2002), heading time (Tanio and Kato, 2007), and kernel hardness (Lesage et al., 2012). Nevertheless, we are not aware of any prior report on the transfer of genes/QTLs for grain yield (GY) or drought resistance in wheat from either wild or domesticated donor lines.

The potential of wild emmer wheat for improving drought resistance in domesticated wheat has been explored in previous studies in our lab. A wide collection of wild emmer wheat populations, originating from across an aridity gradient in Israel, revealed extensive genetic diversity for drought responses in terms of productivity and related drought-adaptive traits (Peleg et al., 2005), as well as high DNA polymorphism (Peleg et al., 2008a). A considerable number of wild accessions exhibited superior performance under drought as compared to durum wheat control cultivars. The greatest allelic diversity and highest drought-resistance potential were observed in wild emmer populations from intermediate aridity levels exposed to the widest climatic fluctuations. Subsequently, a recombinant inbred line population derived from a cross between durum wheat (cv. Langdon) and wild emmer (acc. G18-16) was used to map QTLs conferring drought resistance and related traits (Peleg et al., 2009). Out of 110 mapped QTL, in 58 QTLs the wild emmer allele showed an advantage over the domesticated one. Several QTLs interacted with the environmental conditions and accounted for productivity and related traits under either drought treatment (20 QTLs) or a well-watered control treatment (15 QTLs), or in terms of drought-susceptibility index (S) (22 QTLs).

In the current study, we examined the hypothesis that introgression of selected genomic regions from wild emmer wheat can enhance drought resistance in modern durum and bread wheat cultivars. Our result provide the first evidence that introgression of QTL alleles from wild emmer wheat can enhance productivity and drought resistance in domesticated wheat.

## Materials and methods

### Development of near-isogenic lines

A marker-assisted backcross program (Figure S1) was employed for the introgression of the target regions into elite Israeli durum wheat (cvs. Inbar and Uzan) and bread wheat (cvs. Bar Nir and Zahir). Genomic regions in which the wild QTL alleles showed an advantage over the domesticated alleles in plant productivity, particularly under drought, or in susceptibility indices, were selected based on a previous mapping study (Peleg et al., 2009) for introgression. No significant two-locus epistasis was found between any of the QTLs controlling any of the mapped traits. Marker-assisted backcrossing was based on SSR markers flanking and within each of the target QTL regions (Table 1). In cases where markers were not polymorphic between the recurrent parent and the wild donor or in cases of DArT markers, additional SSR markers were selected from our map (Peleg et al., 2008b) or from other published maps (Somers et al., 2004; Zhang et al., 2008) and integrated into our target region using genetic mapping package *MultiPoint* (MultiQTL Ltd. Haifa, Israel). The length of the target regions varied from 41 to 57 cM, whereas the flanking markers used for selection were 48–61 cM apart. Extraction of genomic DNA was based on Doyle and Doyle (1990). PCR amplification of SSR markers followed Peleg et al. (2008b), fragment analysis was conducted using an automated sequencer (3130XL, Applied Biosystems, Foster City, CA) and analyzed with Peak Scanner Software version 2.1 (Applied Biosystems).

**Table 1:** **Drought-related QTL regions targeted for introgression from wild emmer wheat acc. G18-16 into bread and durum wheat cultivars, SSR markers used for selection, and recurrent backgrounds of the introgression lines**.

**Chromosome and flanking markers (cM)**	**Details of target region^a^**			**Markers used for selection^c^**	**Recurrent backgrounds**
	**QTL^b^**	**LOD**	**PEV**		
Chr.1BL *Xgwm*18 (74.0), *Xgwm806* (130.7)	GY-S	3.3	9.9	*Xgwm18* (74.0), *Xgwm748*(107), *Xgwm806* (130.7)	Inbar, Uzan
Chr.2BS *XwPt-8097* (38.4), *XwPt-6576* (81.9)	GY	11.9	13.1	*Xgwm*1128 (24.8), *Xwmc35* (64.6), *Xgwm1177* (78.9)	Inbar, Uzan
	HI	19.4	12.8		
Chr.7AS *Xgwm60* (0), *XtPt-1755* (46)	SpDM-d	4.4	7.3	*Xgwm60* (0), *Xwmc422* (51.0) / *Xwmc596* (48.0)^d^	BarNir, Zahir, Inbar
	TotDM-d	3.3	9.0		
Chr.7BS (*XwPt-8920* (0), *Xgwm537* (40.7)	GY-d	8.6	12.7	*Xgwm569* (0), *Xgwm537* (61.12)	Inbar
	SpDM	9.0	12.5		
	HI	14.1	22.4		

a *Target region locations, QTLs, Log of likelihood (LOD) and percent of variation explained (PEV) are based on Peleg et al. (2009)*.

b *QTLs conferred grain yield (GY), harvest index (HI), spike dry matter (SpDM) and total dry matter (TotDM) under drought (−d), as a susceptibility index (−S), or across the two treatments (no letter appended)*.

c *SSR markers selected from our map (Peleg et al., 2008b) or from other published maps (Somers et al., 2004; Zhang et al., 2008) were used for selection*.

d *Marker Xwmc422 was not polymorphic between the wild donor and cv. Zahir, Xwmc596 was used as replacement*.

For the development of near-isogenic lines (NILs), selected recombinant inbred lines containing wild donor (G18-16) alleles in the target regions with ~50% domesticated backgrounds (cv. Langdon) were crossed as female parents with the recurrent cultivars (Figure S1). The resultant F_1_ plants were subsequently backcrossed as male parents with the recurrent cultivars as females to restore the recurrent cytoplasmic genomes. BC_1_F_1_ (and subsequently BC_2_F_1_) plants with heterozygous genotype in the target regions were selected by SSR markers and then backcrossed as female lines with the recurrent parent to produce BC_3_F_1_ plants, expected to contain ~6% of the donor recombinant inbred line chromatin (~3% wild chromatin). BC_3_F_1_ with heterozygous genotype in the target regions were self-fertilized to produce BC_3_F_2_ progenies from which homozygous genotypes for the wild allele were selected using molecular markers and selfed to produce BC_3_F_3_ NILs. In addition, BC_3_F_2_ progenies homozygous for the recurrent parent alleles in the target region were selfed to obtain near-isogenic controls (NICs). The developed lines were designated as: NIL/NIC-P-Chr-#, where P is the first letter of the recurrent parent's name (B, I, U, or Z), Chr is the chromosome containing the introgression, and # is the line number.

Finally, the parental lines and most NILs and NICs were genotyped using the 15K array (TraitGenetics GmbH, Gatersleben, Germany) containing ~13,000 markers that have been selected from the wheat 90K array (Wang et al., 2014). The genotypes of our lines were aligned with the recently published high density tetraploid wheat consensus map integrating SSR, DArT, and SNP markers (Maccaferri et al., 2015).

### Growth conditions

**Figure 1:**
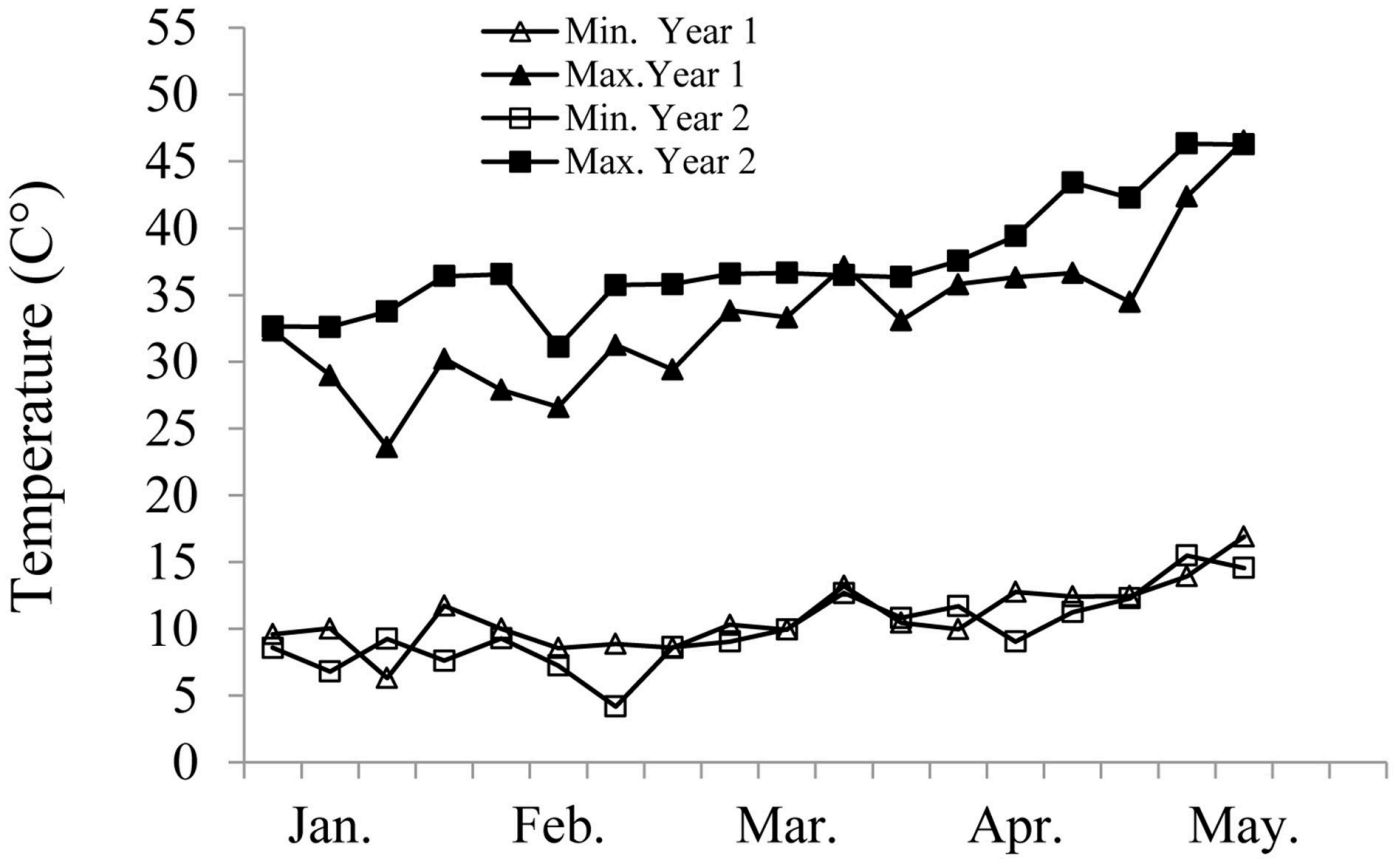
**Average weekly minimum and maximum temperatures measured in the screen house in Year 1 and Year 2**.

The resultant NILs and NICs, as well as their recurrent parents, the wild donor line (G18-16) and domesticated parent of the mapping population (cv. Langdon), were evaluated for their drought responses during the winters of 2012–13 (Year 1, BC_3_F_3_) and 2013–14 (Year 2, BC_3_F_4_). Most of the genotypes were examined across the two years, excluding a few that were available and tested only in Year 2. Seeds were placed in moist germination paper for two weeks in a dark cold room (4°C), followed by 3 days acclimation at room temperature (22°C). The seedlings were then transplanted into an insect-proof screen house protected by a polyethylene top at the experimental farm of The Hebrew University of Jerusalem in Rehovot, Israel (31°54′N, 34°47′E; 54 m above sea level). The soil at this location is brown-red degrading sandy loam (Rhodoxeralf), composed of 76% sand, 8% silt, and 16% clay (w/w). A split-plot factorial (genotype x irrigation regime) block design with five replicates was employed; each block consisted of two main plots (for the two irrigation regimes), splited into subplots for genotypes. Each subplot consisted of a single row, with five plants, 10 cm apart (50-cm long plots). Two 40-cm spaced rows were planted on each bed, with 100 cm between each pair of rows. The field was treated with fungicides and pesticides as needed, and was weeded manually once a week. Two irrigation regimes were applied via a drip system: well-watered control (WW) and water-limited (WL). To mimic the natural pattern of rainfall in the eastern Mediterranean region, water was applied during the winter months starting from planting (23 December 2012 and 19 December 2013 for Years 1 and 2, respectively) and ending in early spring (31 March 2013 and 23 March 2014, respectively) for the WL treatment, or later (28 Apr 2013 and 20 Apr 2014, respectively) for the WW treatment. The WW treatment was irrigated twice a week, whereas the WL treatment was irrigated twice every other week. The total seasonal water application was 710 and 670 mm for the WW treatment, and 360 and 290 mm for the WL treatment, in Years 1 and 2, respectively. Weekly average temperatures in the screen house varied between a minimum of 4–18°C and a maximum 24–47°C (Figure 1). The number of hot days (>40°C) recorded in Year 1 was 12 with the first occurrence in March, and 23 in Year 2 with first occurrence in January.

### Phenotypic measurements

Heading time, defined as the date at which the first spike of 50% of the plants in a plot was fully exposed, was recorded based on daily inspection and used to calculate days from planting to heading (DP–H). Culm length (CL) was measured at maturity from the soil surface to the base of the three first spikes per plot. Osmotic potential (OP) was measured on 7 March and 16 February for Years 1 and 2, respectively. Flag-leaf blades were sampled at dawn, placed in plastic tubes with their bases dipped in distilled water and kept for 6 h in a dark cold room (4°C) for full rehydration. Leaves were removed from the water, blotted with a paper towel, placed in plastic test tubes, frozen in liquid nitrogen and kept at −18°C until measurement. The leaves were defrosted, the tube was pierced with hot needle, placed in a bigger test tube and centrifuged (15 min, 10,0000RPM) to obtain leaf sap. OP of the leaf sap was assessed using a vapor pressure osmometer (model 5520; Wescor Inc., Logan, UT, USA). Osmotic adjustment (OA) was calculated as the difference between OP in the individual WL plots and the genotypic mean in the WW treatment (Blum, 1989). At full grain maturity, all aboveground biomass was harvested; spikes were counted to assess the number of fertile spikes per plant (Sp/P), separated from the vegetative organs (stems and leaves), and both were oven-dried (80°C or 38°C for 48 h for vegetative organs and spikes, respectively) and weighed. Spike dry matter and total dry matter (TotDM) were determined. Samples (20 and 40 g for Years 1 and 2, respectively) of spikes from each plot were then threshed and used to calculate grain yield (GY), harvest index (HI = GY/TotDM), grain number per spike (G/Sp) and thousand grain weight (TGW).

### Statistical analyses

Drought susceptibility index (S) was calculated for TotDM and GY according to Fischer and Maurer (1978) as: S = (1−Y_WL_/Y_WW_)/(1−X_WL_/X_WW_), where Y_WL_ and Y_WW_ are the mean performances of a specific genotype under the respective treatments, and X_WL_ and X_WW_ are the mean performances of all genotypes under those treatments.

The JMP version 7.0 statistical package (SAS Institute, Cary, NC, USA) was used for statistical analyses. A factorial model was employed for the analysis of variance (ANOVA), conducted separately for each recurrent parent and its derivative lines, with blocks as random effects and irrigation regime and genotype as fixed effects. Mean values of each NIL were compared with those of its recurrent parent and NIC (where available). The wild donor line (G18-16) and the mapping population domesticated parent (cv. Langdon) were presented as references without a statistical analyses.

The associations among the traits reflecting productivity and drought responses were examined using correlation analyses and principal component analysis (PCA). Two components were extracted using eigenvalues >1 to ensure meaningful implementation of the data by each factor.

Heritability estimates (*h*^2^) were calculated for each trait under each irrigation treatment using a linear regression coefficient (b) between BC_3_F_3_ (Year 1) and their BC_3_F_4_ progenies (Year 2) (Cahaner and Hillel, 1980).

## Results

### Main effects of genotypes, water regimes, and years

Four genomic regions (Table 1) were successfully introgressed (out of six initially targeted regions), three of them into one or two durum genetic backgrounds and one into two bread wheat and one durum background, resulting in a total of eight target x background combinations, with two to four sister NILs per combination. Out of the ~13,000 SNP markers used to genotype the introgression lines and their parents, 7880 markers were aligned with the two sub-genomes of the high density tetraploid wheat consensus map (Maccaferri et al., 2015). Out of these, 4400–4800 (56–61%) SNPs exhibited polymorphism between the donor parental lines (G18-16 and Langdon) and their respective recurrent parent. In accordance with the expected rate of donor chromatin (6%), between 3.3% and 9.5% of polymorphic markers were identified as introgressed from either G18-16 or Langdon, including the targeted genomic regions. The results of the SNP genotyping provided a high resolution confirmation for the introgressed genomic regions. SNP genotyping of selected NILs, containing the 2BS QTL in the background of durum wheat cv. Uzan and 7AS QTL in the background of bread wheat cv. Bar Nir, as well as their respective parental genotypes are presented in Table S1.

ANOVA carried out separately for each of the recurrent cultivars and their derivative lines revealed highly significant effects of genotype (G) and irrigation treatment (I) on most traits in both years (Tables S2, S3). GxI interactions were only significant in a few cases, at a lower significance levels than those of the main effects. Potential plant productivity, averaged across all genotypes under WW treatment, was 25.6 g for GY and 50.6 g for TotDM in Year 1, and 17.9 and 38.5 g, respectively, in Year 2. The lower productivity in Year 2 presumably reflects the impact of higher temperatures (Figure 1) and abundance of heat spells in that year. The average GY and TotDM production under the WL treatment was ~51 and ~50% of the WW control in Year 1 and Year 2, respectively, suggesting a similar magnitude of water stress in both years.

Heritability estimates were calculated based on parent–offspring correlation (between years) for durum and bread wheat separately; about half of the estimates were found statistically significant and varied between 0.53 and 0.99 (Table 2), indicating a stable genotypic ranking across the 2 years. The highest heritability estimates across the two treatments were obtained for GY, TotDM, and DP–H in both bread and durum wheat.

**Table 2:** **Estimated heritability for the measured traits: total dry matter (TotDM), grain yield (GY), harvest index (HI), spikes per plant (Sp/P), grains per spike (G/Sp), thousand grain weight (TGW), days from planting to heading (DP–H), culm length (CL), and osmotic potential (OP), for each ploidy level under WW, well-watered; WL, water-limited treatments**.

**Trait**	**Heritability estimates**			
	**Bread wheat (*n* = 7)**		**Durum wheat (*n* = 17)**	
	**WW**	**WL**	**WW**	**WL**
GY	0.85^*^	0.99^***^	0.45	0.72^***^
TotDM	0.92^**^	0.91^**^	0.64^**^	0.72^**^
HI	0.35	−0.39	0.56^*^	0.69^**^
Sp/P	0.88^**^	0.89^**^	0.42	0.44
G/Sp	0.50	0.68	0.17	0.61^**^
TGW	0.25	0.82^*^	0.81^***^	0.53*
DP–H	0.80^*^	0.73	0.82^***^	0.92^***^
CL	0.96^***^	0.95^**^	−0.14	0.10
OP	0.20	0.66	0.37	0.50^*^

*, **, *** *P < 0.05, 0.0,1 and 0.001, respectively, for the calculated linear regression coefficient (b)*.

### Performance of NILs by genetic background

**Figure 2:**
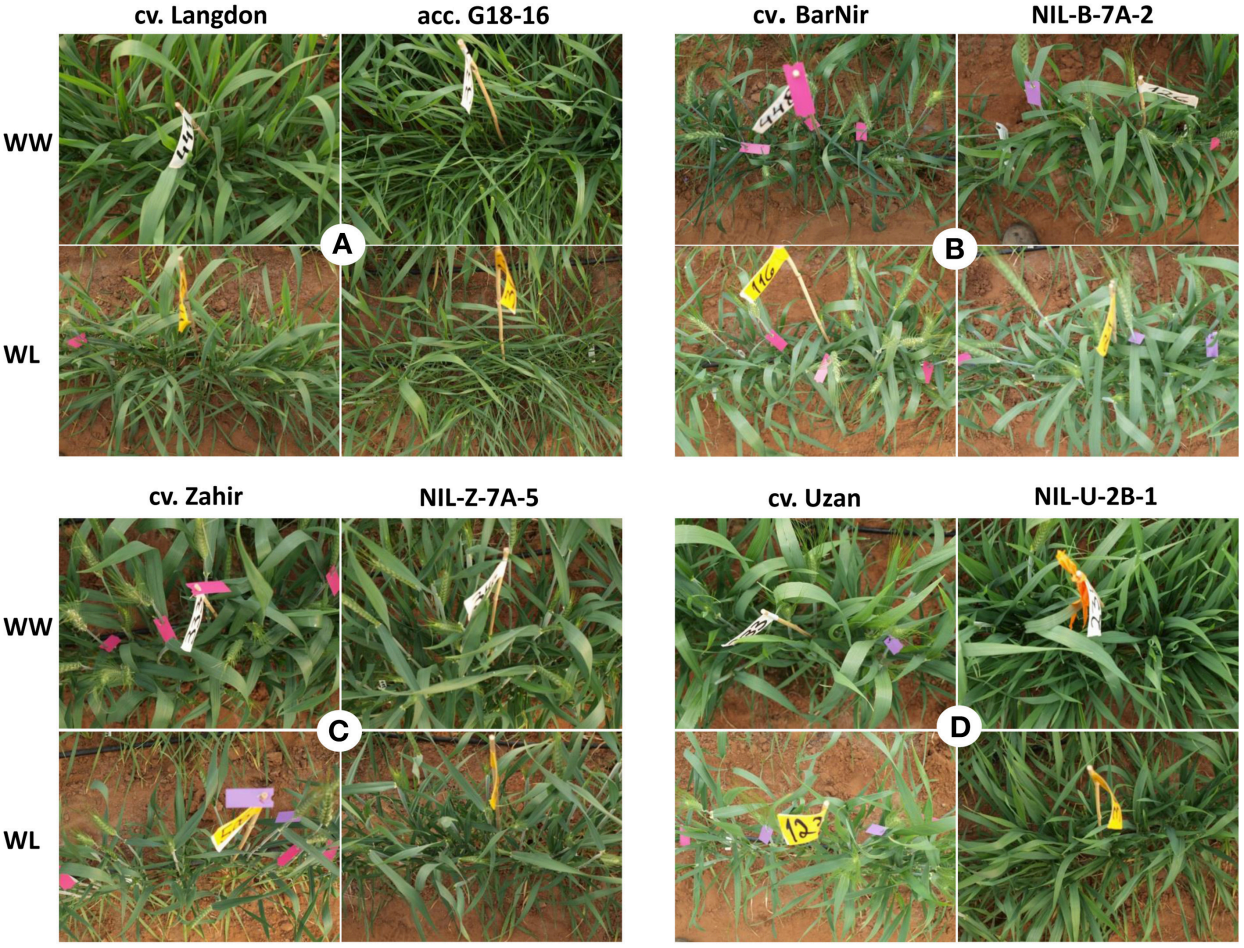
**Photographs of typical experimental plots of mapping population parental lines (A), recurrent parents and their derivative NILs (B–D) in Year 1 under the well-watered (WW) and water limited (WL) treatments**.

The wild donor line (G18-16) exhibited TotDM similar to the recurrent cultivars, ~50% lower GY and 8–18 days later heading (Table 3, Figure 2A), whereas domesticated parent of the mapping population (cv. Langdon) exhibited about double TotDM, similar GY and 24–34 days delayed heading compared to the recurrent parents.

**Table 3:** **Total DM (TotDM), grain yield (GY), and days from planting to heading (DP–H) under well-watered (WW) and water-limited (WL) treatments and susceptibility index for TDM and GY in Years 1 and 2**.

**Chro-mosom**	**Genotype**	**SSR alleles**	**GY (g/plant)**						**TotDM (g/plant)**						**DP-H**			
			**Year 1**			**Year 2**			**Year 1**			**Year 2**			**Year 1**		**Year 2**	
			**WW**	**WL**	**S**	**WW**	**WL**	**S**	**WW**	**WL**	**S**	**WW**	**WL**	**S**	**WW**	**WL**	**WW**	**WL**
	G18-16		11.9	5.3	1.15	7.5	2.7	1.31	51.3	24.9	0.99	41.4	14.2	1.35	81	81	78	84
	Langdon		29.1	11.7	1.24	23.5	9.2	1.23	96.0	44.7	1.03	76.7	30.7	1.23	96	94	97	100
Recurrent parent - Bread cv. Bar Nir			23.5	11.8	1.03	18.0	7.3	1.21	42.2	22.5	0.90	34.7	13.2	1.28	64	63	63	62
Chr.7AS	NIL-B-7A-1	G–G	28.8	15.4	0.97	19.6	9.7	1.03	50.1	27.5	0.87	37.9	18.4^*^	0.85^*^	61^***^	61^*^	61^*^	59^**^
	NIL-B-7A-2	G–G	28.0	18.8^*^	0.68^**^	16.1	11.8^*^	0.60^**^	50.9	34.1^*^	0.63^*^	33.3	22.1^*^	0.67^***^	66^***^	65^***^	67^***^	66^***^
	NIL-B-7A-3	P–G				21.8	10.6	1.04				41.0	18.5	1.13			64	62
	NIC-B-7A-2	P–P				12.4^*^	8.1	0.71				35.3	17.8	0.97			68^***^	64^*^
Recurrent parent - Bread cv. Zahir			21.0	12.9	0.80	15.8	8.1	1.02	39.8	23.7	0.78	29.5	14.1	1.10	64	62	61	59
Chr.7AS	NIL-Z-7A-2	G–P	18.3	8.4	1.13	12.5	5.3	1.16	33.5	17.7	0.91	24.2	9.7	1.23	63	66^**^	60	59
	NIL-Z-7A-5	G–P	29.8^**^	17.0	0.94	21.1^*^	11.0	0.94	54.6^**^	33.3	0.79	41.2^*^	20.5	1.00	71^***^	71^***^	67^***^	67^***^
	NIL-Z-7A-4	P–G	18.8	10.2	0.96	15.5	7.7	1.01	32.8	17.8	0.88	30.1	14.8	1.04	64	62	64^*^	60
Recurrent parent - Durum cv. Inbar			29.0	12.3	1.20	22.8	10.0	1.14	55.5	26.3	1.01	45.0	18.7	1.20	72	72	75	72
Chr.1BL	NIL-I-1B-1	G-G-P	21.2^**^	9.4	1.16	14.7	7.5	0.99	39.5^**^	19.5	0.97	28.4^*^	15.0	0.97	63^***^cc	64^***^	67^***^	63^***^cc
	NIL-I-1B-2	G-G-P	18.9^*^	13.3	0.79	18.9	9.2	1.04	23.4^**^	16.6	0.60^*^	24.7	12.2	1.11	65^***^	66^***^	70^***^	64^***^cc
	NIC-I-1B-1,2	P-P-P	26.8	14.2	0.98	21.0	10.9	0.98	52.7	28.7	0.88	41.9	20.6	1.04	67^***^	67^***^	68^***^	67^***^
Chr.2BS	NIL-I-2B-1	G-G-G	28.0	13.8	1.05	13.1^**^	9.9	0.49^**^	52.9	29.3	0.86	28.7^*^	20.5	0.59^*^	71	70	71^*^	68^**^
	NIL-I-2B-2	G-G-G	30.6	10.3	1.38c	14.4^**^	8.0	0.90	59.1	24.2	1.14c	28.8^*^	17.2		73	71	74	72
	NIL-I-2B-3	G-P-P	23.5^*^	12.6	0.97	18.2	9.5	0.96	44.7^*^	26.6	0.78	36.2	19.6	0.94	72	72	71^*^	68^**^
Chr.7AS	NIL-I-7A-1	G–G	33.7	14.2	1.21	22.0cc	12.3	0.90	62.4	28.5	1.04	43.8c	25.1	0.88	72c	72	75	73
	NIL-I-7A-2	G–G	27.8	12.1	1.17	20.4cc	12.2	0.82	49.6	25.0	0.95	39.2	26.1	0.91	70^*^	68^***^	62c	62
	NIC-I-7A-1	P–P	41.7^***^	16.6	1.25	30.6^**^	15.2	1.02	73.5^***^	33.4	1.05	61.4^**^	29.3	1.07	76^**^	73	77	72
Chr.7BS	NIL-I-7B-1	G–G	26.9	12.2	1.15	20.8	7.9	1.26	54.7	26.2	1.00	43.9	17.1	1.25	66^***^c	66^***^cc	70^***^	68^**^
	NIL-I-7B-2	G–G	20.8**cc	12.3	0.85*c	23.5	8.7	1.28	40.4**cc	26.8	0.65**cc	48.5	16.5	1.35	61^***^ccc	66^***^cc	66^***^	60^***^
	NIC-I-7B-1,2	P–P	29.7	11.5	1.28	19.8	8.3	1.18	58.9	25.9	1.08	43.6	18.7	1.17	70	70	69^***^	68^***^
Recurrent parent - Durum cv. Uzan			26.1	11.4	1.17	13.3	4.3	1.37	47.9	23.4		25.6	9.6	1.29	67	67	67	65
Chr.1BL	NIL-U-1B-1	G-G-G	25.0	13.3	1.22	11.2	11.0^*^	0.50^**^	48.0	24.4	0.70^*^	32.1	25.9	0.54^*^	76^***^	73^***^	69	73^***^
	NIL-U-1B-2	G-G-G	23.0	12.9	0.92	18.0	6.1	1.46	46.1	29.4	0.63^**^	45.5^**^	15.1	1.42	74^***^	73^***^	68	65
	NIL-U-1B-3	G-G-G				15.1	6.8	1.12				31.8	15.0	1.05			68	65
	NIL-U-1B-4	G-G-P				11.2	10.5	0.12^**^				28.0	23.7	0.68^*^			70	67
Chr.2BS	NIL-U-2B-1	G-G-G	25.6^**^	14.8^*^	0.96	26.0^***^	13.8**ccc	1.13	65.9^***^	36.9^*^	0.86	61.3^***^	34.2^***^	1.07	95^***^	92^***^	89^***^	92^***^
	NIL-U-2B-2	P-G-G				19.6^*^	8.3	1.17				43.4^**^	20.0	1.11			72^*^	69
	NIL-U-2B-3	P-P-G				19.7^*^	11.0^*^	0.89				40.0^*^	21.5	0.98			68	67
	NIC-U-2B-3	P-P-P				13.5	7.8	1.21^**^				30.9	15.2	1.09			68	65

Mean comparisons by t-test between each of the lines and its recurrent parents (

*, **, *** *) or its near isogenic control (NIC, c, cc, ccc) under a specific irrigation treatment at P < 0.05, 0.01, and 0.001, respectively. SSR alleles, G-wild emmer wheat; P-parental cultivar*.

#### Bread wheat cv. Bar Nir and derivatives

**Figure 3:**
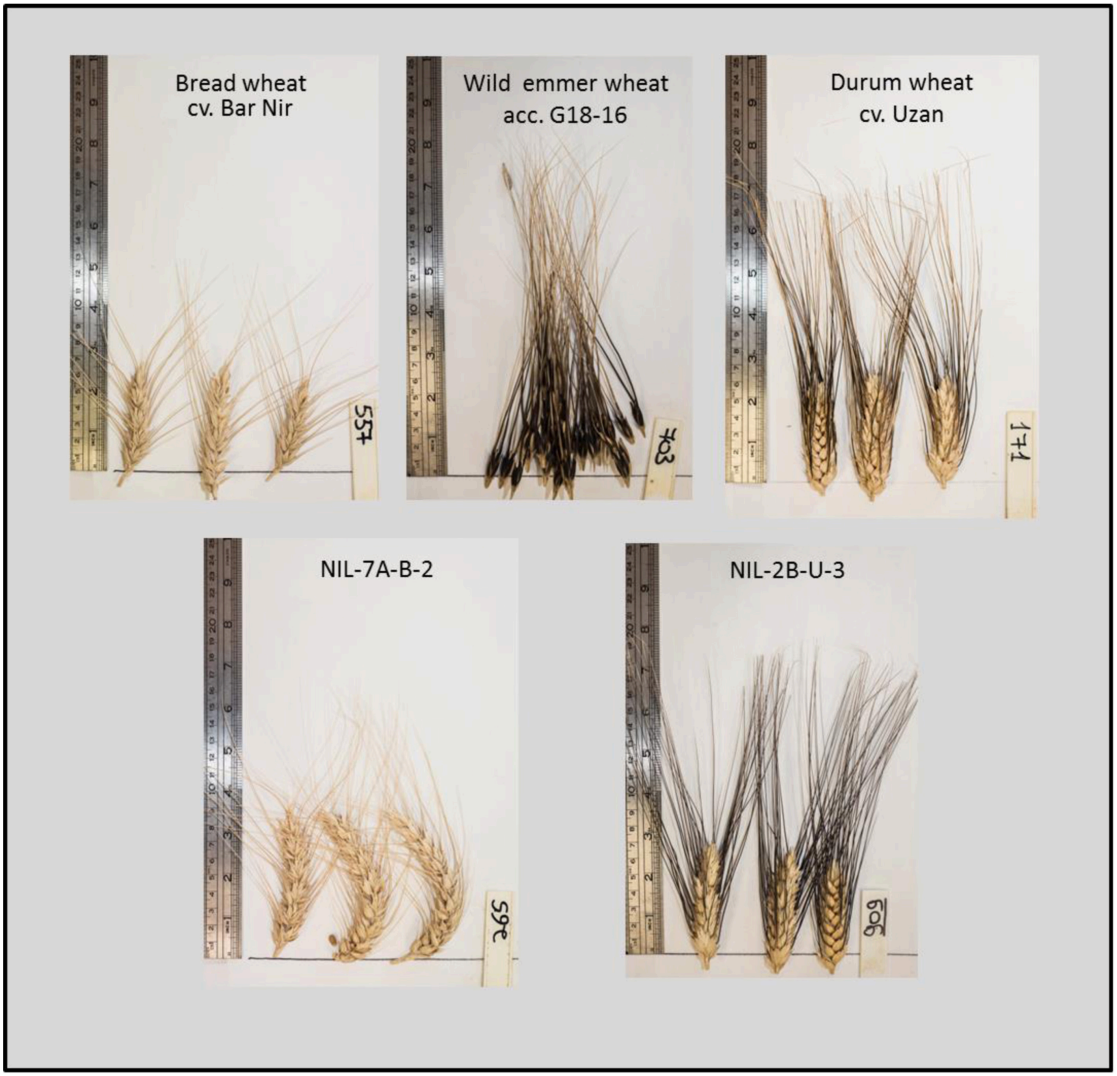
**Three first spikes of the wild emmer wheat donor acc**. G18-16 (articulated) the recurrent parents bread wheat Bar Nir and durum wheat Uzan and their derivative NILs, grown under water-limited treatment, 2013-14.

NILs that carry the introgression from wild emmer on Chr 7AS in the background of bread wheat cv. Bar Nir showed the most promising results. NIL-B-7A-2 exhibited a significant advantage (60%) consistently across the 2 years over the recurrent parent for both GY and TotDM under the WL treatment (Table 3, Figure 2B). Zooming in on yield components of NIL-B-7A-2 under drought showed an advantage over the recurrent parent in Sp/P and G/Sp (Table S4, Figure 3) across the two years, although it did not meet the common statistical threshold (Table S4). Similar trends were observed in NIL-B-7A-1 and NIL-B-7A-3 (the latter containing a partial introgression), although in most cases they were not statistically significant. NIC-B-7A-2, a sister line of NIL-B-7A-2, did not differ from the recurrent parent, “Bar Nir,” thus supporting the assumption that the superior performance of the latter (NIL-B-7A-2) is governed by the introgressed region. The phenology (DP-H) of these NILs and NIC was slightly and inconsistently modified (significant; Table 3), whereas CL exhibited higher values in most lines, with the exception of NIL-B-7A-3, which was similar to the recurrent parent (Table S5). Interestingly, NIL-B-7A-2 exhibited also a significantly lower OP under drought and greater OA, with similar trends observed in NIL-B-7A-3.

#### Bread wheat cv. Zahir and derivatives

Three NILs were introgressed with the Chr 7AS target region from wild emmer in the background of bread wheat cv. Zahir, each containing a segment of the target region. NIL-Z-7A-5, which was introgressed with the upper segment of the 7AS target region, exhibited between 32 and 45% advantage over the recurrent parent in GY and TotDM (significant for WW and nearly significant for WL) coupled with 6–9 days delayed DP-H, consistently across the two years (Table 3, Figure 2C). NIL-Z-7A-2, also introgressed with the upper segment of the 7AS target region, as well as NIL-Z-7A-4, introgressed with the lower segment, largely resembled the recurrent parent both in productivity and phenology.

#### Durum wheat cv. Inbar and derivatives

Four target regions (on Chr 1BL, 2BS, 7AS, 7BS) were successfully introgressed into the background of durum wheat cv. Inbar. Nevertheless, none of these introgressions was associated with improved productivity in the resultant NILs (Table 3). On the contrary, in several cases, significantly lower productivity, in terms of GY or TotDM, was recorded. Surprisingly, NIC-I-7A-1, which according to the SNP genotyping contains a small (8cM) unintentional introgression at the lower end of the 7AS target region (and beyond), presented significantly higher GY and TotDM in both years under WW conditions.

Among the three yield components studied, in most cases, G/Sp was significantly reduced relative to the recurrent parent (Table S4). Introgression of the Chr 1BL and 7BS QTLs induced 5- to 10-day earlier heading in the resultant NILs, as well as in the respective NICs, albeit to a lesser extent (Table 3). The NILs introgressed with Chr 2BS QTLs exhibited lower OP under the WL treatments and greater OA capacity in Year 2.

#### Durum wheat cv. Uzan and derivatives

Two target regions (on Chr 1BL and 2BS) were successfully introgressed into the background of durum wheat cv. Uzan, and both showed significantly improved performance relative to the recurrent parent (Table 3).

Among the NILs carrying the 1BL target region introgressed in the background of cv. Uzan, NIL-U-1B-1 exhibited greater GY and TotDM under drought and a lower S (greater stability across environments) for both variables in Year 2, whereas NIL-U-1B-4 exhibited similar trends which were only significant for S values. In these two lines, the improved GY in Year 2 was associated with greater Sp/P, G/Sp (Figure 3) and TGW, although these effects were only significant in a few cases (Table S4). Compared to the recurrent parent, heading time of NIL-U-1B-1 was delayed by ~6 days (averaged across the two years and treatments), whereas it was not significantly delayed in NIL-U-1B-4 (Table 3). Both NILs exhibited a slight increase in CL, which was significant in only a couple of cases.

For the 2BS target region, three NILs were developed (NIL-U-2B-1, 2, and 3), each containing a different segment of the target (3, 2, and 1 markers, respectively), as well as one NIC. NIL-U-2B-1, containing the entire target region (3 markers), consistently exhibited the highest GY and TotDM among the three NILs across years and treatments, accompanied by a 25 day-delayed heading time (Table 3, Figure 2D), which makes it unsuitable for meaningful comparison to the recurrent parent. The two other NILs, tested only in Year 2, also exhibited improved productivity across the two treatments without a major modification in their phenology. NIC-U-2B-3, a sister line of NIL-U-2B-3 and also related to the other two NILs, did not differ from the recurrent parent, ‘Uzan,’ thus validating the effects of the introgressed QTL region. All three NILs exhibited greater (and usually significant) Sp/P and TGW compared to the recurrent parent (Table S4).

### Association between productivity and related traits

**Figure 4:**
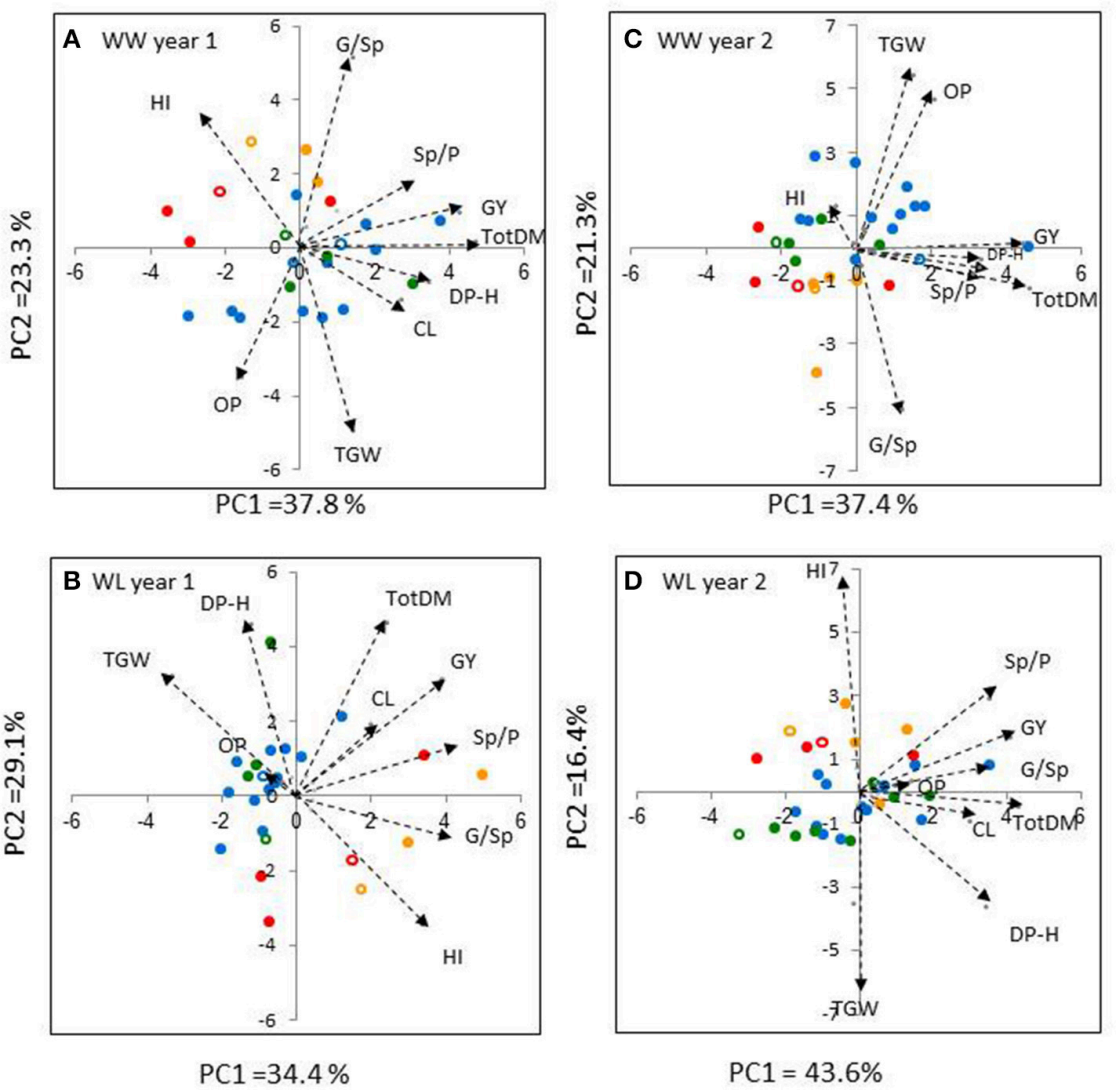
**Principal component analysis (PCA) based on correlation matrix of traits: (TotDM), total dry matter; (GY), grain yield; (HI), harvest index; (Sp/P), spikes per plant; (G/Sp), grains per spike; (TGW), thousand grain weight; (DP–H), days from planting to heading; (CL), culm length; fertile spikes per plant, (OP), osmotic potential; recorded on 25 and 32 genotypes in Year 1 (A,B) and Year 2 (C,D), respectively, under well-watered (WW), and water-limited (WL) irrigation regimes**. Biplot vectors are trait factor loadings for PC1 and PC2. Each of the recurrent cultivar groups is colored differently (cv. Inbar–blue, cv. Uzan–green, cv. Bar Nir–orange, cv. Zahir–red). Recurrent cultivars are indicated by open symbols.

The PCA analyses of the introgression lines and recurrent parents (*n* = 25 and 32 in Years 1 and 2, respectively) extracted two major principal components (eigenvalues >1) that accounted, collectively between 58.7 and 63.5% of the variance for each treatment x year combination (Figure 4). All four PCAs revealed a fairly clear separation between the durum wheat genotypes (in blue or green) and the bread wheat genotypes (in orange or red). Under the WW treatment, PCAs exhibited similar trends across years (Figures 4A,C); PC1 (X-axis) was positively loaded with TotDM, GY, Sp/P, and DP–H, whereas PC2 (Y-axis) was loaded with G/Sp and at the opposite direction with TGW and OP. In contrast, under the WL treatment, mixed trends were observed across years.

Correlation analyses of GY vs. yield components and physiological traits were conducted separately for durum and bread genotypes in each of the four environments. In most cases, GY exhibited highly significant positive correlations with both TotDM and Sp/P (Table 4) in agreement with the similar directions of these traits' vectors in all four PCAs (Figure 4). Among the two other yield components, G/Sp correlated positively with GY only in two cases in the durum genotypes, whereas TGW did not show any significant correlation to GY.

**Table 4:** **Correlation of various traits with grain yield (GY) under the different treatments and years**.

**Trait**	**Confidents of correlation with GY**							
	**Bread wheat**				**Durum wheat**			
	**Year 1 (*n* = 7)**		**Year 2 (*n* = 9)**		**Year 1 (*n* = 17)**		**Year 2 (*n* = 22)**	
	**WW**	**WL**	**WW**	**WL**	**WW**	**WL**	**WW**	**WL**
TotDM	0.99^***^	0.98^***^	0.81^**^	0.96^***^	0.86^***^	0.67	0.89^***^	0.90^***^
HI	−0.13	0.50	0.43	0.08	0.04	0.05	0.33	0.38
Sp/P	0.66	0.82^*^	0.68^*^	0.83^**^	0.74^***^	0.57^*^	0.86^***^	0.92^***^
G/Sp	0.63	0.70	−0.03	0.22	0.51^*^	0.32	0.37	0.60^**^
TGW	0.20	−0.13	0.06	0.15	0.10	0.48	0.12	0.04
DP–H	0.43	0.30	0.05	0.66	0.29	0.47	0.46^*^	0.53^*^
CL	0.70	−0.14	−0.11	0.19	0.38	0.25	0.69^***^	0.73^***^
OP	−0.52	0.61	0.34	−0.57	−0.17	0.21	0.49^*^	0.55^**^
OA	−0.27	−0.29	0.44	0.45	−0.15	−0.21	−0.15	−0.47^*^

*, **, *** *P < 0.05, 0.01, and 0.001, respectively*.

## Discussion

QTL mapping is a widely accepted approach to dissect quantitative traits into their single genetic determinants and relating phenotypic differences to their genetic basis (Paterson, 1995; Tuberosa and Salvi, 2006; Collins et al., 2008). A large number of QTL studies have been published in the last few decades on various traits in crop plants, including wheat. Nevertheless, the number of studies attempting to validate mapped QTLs and explore their potential for crop breeding is very small, particularly with respect to complex polygenic traits such as yield and responses to abiotic-stresses (Cattivelli et al., 2008; Levi et al., 2009; Salvi and Tuberosa, 2015). Therefore, the current study provides a unique opportunity to evaluate the potential of QTL introgression via marker assisted selection for the improvement of such traits.

### Validation of the introgressed QTL alleles

*The Chr 7AS target region*, in which the wild allele conferred, in our previous mapping study (Peleg et al., 2009), higher GY and TotDM exclusively under WL conditions (inductive QTLs), was introgressed into three genetic backgrounds. NILs introgressed with this region in the background of durum wheat cv. Inbar did not manifest improved productivity (Table 3). Nevertheless, NIC-I-7A-1, containing a small unintentional introgression at the lower end of the 7AS target region, exhibited improved productivity in both years under WW conditions. This finding suggests that cv Inbar can be improved by wild emmer allele introgressions, which is however subjected to QTL × background and G × E interactions.

In the background of bread wheat cv. Bar Nir, SNP genotyping showed two separate introgressions within the Chr 7AS target region (Table S1), which might have resulted from a double recombination that was not identified due to absence of central marker for selection. Nevrtheless, NILs introgressed with this regions into the background of Bar Nir exhibited a pronounced improvement in GY and TotDM production, which was found to be significant only under WL treatment (Table 3, Figure 2B), thus validating the QTL X environment interaction. Introgression of the upper part of this genomic region into the background of cv. Zahir (NIL-Z-7A-5) also improved plant productivity, which was found to be significant under WW treatment and somewhat below significance threshold in WL treatment (Figure 2C). NIL-Z-7A-2, selected for the same introgression did not exhibit such advantage, presumably because of a modified introgression which was not traced (SNP genotyping not available).

The 7AS genomic region seems to play a major role with respect to productivity and stress responses in wheat. A large number of studies, using a wide variety of genetic materials and populations, have found a large number of QTLs for numerous traits in the 7AS chromosome arm, including plant productivity, yield components, phenology, morphology, physiology and metabolites (Table S6 and references therein).

QTLs for drought (Quarrie et al., 2006; Bennett et al., 2012; Hill et al., 2013) and salinity (Shavrukov et al., 2011) tolerance, two related abiotic stresses (Munns, 2002), were also mapped to the 7AS chromosome arm, thus expanding the scope of this region for improving abiotic-stress resistance in wheat. QTLs associated with several tricarboxylic acid (TCA) cycle intermediates were mapped on 7AS (Hill et al., 2013). Modified TCA-cycle intermediate levels have been associated with OA and drought resistance in cotton (*Gossypium* spp.; Levi et al., 2011). Thus, it is possible that a change in TCA intermediates (not measured in the current study) contributed to the superior OA found in NIL-B-7A-2 and 3 (Table S5).

QTLs for time to heading were previously mapped within the chromosome 7AS target region as well as somewhat above it (Maccaferri et al., 2014 and references therein). It is possible that an introgression of such QTL(s) is responsible for the later time to heading (higher DP-H) observed in several of our 7AS NILs. Nevertheless, heading time of the NILs in the backgrounds of Bar-Nir was only 2–4 days delayed, which enables their comparison to the parental genotypes. Moreover, NIL-B-7A-2 and NIC-B-7A-2 had the same DP-H and yet only the NIL exhibited superior productivity under drought, thus confirming the effect of the introgression regardless of the later phenology.

*The Chr 7BS target region*, in which the wild allele conferred, in our previous mapping study (Peleg et al., 2009), greater GY under drought, higher spike dry matter and HI under both treatments, was introgressed into the background of durum wheat cv. Inbar. The respective NILs (NIL-I-7B-1 and NIL-I-7B-2) exhibited no improvement in yield (Table 3), thus failing to validate the QTL effect. Both NILs exhibited earlier DP-H than their recurrent parent, validating the phenology QTLs mapped in this region by Peleg et al. (2009).

*The Chr 1BL target region*, in which the wild allele conferred, in our previous mapping study (Peleg et al., 2009), lower GY-S (higher stability across environments), was introgressed into two durum wheat backgrounds.

NILs introgressed with this QTL into the background of durum cv. Inbar did not validate the expected phenotype. In the background cv. Uzan, out of the four NILs introgressed with the 1BL target region, two lines (NIL-U-1B-1 and NIL-U-1B-4) exhibited remarkable stability across environments, thus validating the QTL phenotype (Table 3). Moreover, under WL conditions, these two NILs also presented higher productivity in terms of GY and TotDM with no modification in plant phenology, as compared to their recurrent parent, demonstrating this QTL's potential for enhancing wheat performance under drought conditions in certain genetic contexts. This advantage was associated with a greater number of Sp/P and G/Sp, while maintaining a stable TGW (Table S4).

*The Chr 2BS target region*, in which the wild allele was responsible for a higher GY and HI (Peleg et al., 2009), was introgressed into two durum wheat backgrounds (Inbar and Uzan).

In the background of durum cv. Inbar, no improvement in GY or HI was recorded in most cases, whereas in a few cases, significantly lower productivity was observed (Table 3).

In the background of durum wheat cv. Uzan, the set of NILs introgressed with various segments of the 2BS QTL region (Table S1) provide an opportunity to break down the introgressed QTL. NIL-U-2B-1, containing the entire QTL region, exhibited a pronounced advantage in productivity over the parental cultivar (Table 3, Figure 2D), which may reflect the considerably later heading date of the NIL. QTLs for phenology (Hanocq et al., 2004; Maccaferri et al., 2008, 2010) as well as the gene PPD-B1, responsible for a photoperiod-insensitivity (Maccaferri et al., 2014) have been previously mapped in the 2BS chromosome arm using various populations. Such QTLs were not mapped in our previous study (Peleg et al., 2009), suggesting the absence of polymorphism in the respective loci between the two late-heading parental lines (Table 3), in contrast to the earlier heading of the recurrent parent.

Interestingly, both NIL-U-2B-2 and NIL-U-2B-3, which do not contain the wild allele in the upper part of the target region, also exhibited superior performance over the recurrent parent with a similar phenology (Tables 3). The superior yield exhibited by NIL-U-2B-3, containing only the lowest part of the target region, highlights this segment as the locus of interest. The performance of the near-isogenic control (NIC-U-2B-3) was similar to that of the parental genotype and inferior to the three NILs (significant only for NIL-U-2B-1), thus further validating the QTL effect.

The current study validated the Chr 2BS QTLs for GY and CL which were mapped in the same population (Peleg et al., 2009), in agreement with a published QTL for GY (Verma et al., 2004). The respective NILs also exhibited greater TotDM and TGW, the latter in agreement with a published QTL (Kumar et al., 2006).

### Environmental and physiological considerations

Four genomic regions carrying QTLs conferring drought resistance (Peleg et al., 2009) were introgressed from the wild emmer wheat donor line into elite durum or bread wheat cultivars. Three of these introgressions were validated in a specific genetic background: two in the background of durum cultivar Uzan, and the third in the backgrounds of two high-yielding bread wheat cultivars. Moreover, in most of these cases, the QTL X environment interaction (Peleg et al., 2009) was also validated, i.e., an inductive effect specifically expressed under drought for the 7AS QTL, a constitutive effect expressed under both treatments for the 2BS QTL, and a greater stability across the two treatments for the 1BL QTL. The phenotypic effects of the QTL introgressions were usually consistent across the two years, thus lending further support to the validation of the QTL effects. In our previous mapping studies in cotton (Saranga et al., 2001) and wheat (Peleg et al., 2009), partially different sets of QTLs accounted for plant productivity under contrasting water treatments (i.e., WW and WL), suggesting that adaptation to both conditions can be combined in the same genotype. The current validation of QTL X environment interaction reinforces the importance of these results and their applicability. The relatively high heritability estimates of TotDM and GY (Table 2) reflect a high degree of consistency between the two years and a high potential for improvement through selection.

Among the three yield components recorded in our study, Sp/P exhibited the highest correlation with GY consistently across all years, treatments and ploidy levels (Table 4). Moreover, NILs that exhibited an advantage in GY compared to their recurrent parent (Tables 3) also exhibited higher Sp/P (although not always significant, Table S4), a trait that seems to have been contributed by wild donor acc. G18-16 (Figure 2A). An optimal balance between sowing rates and plant tillering can be achieved when using restricted tillering lines in a predictable environment (Donald, 1968); however, results from the current (Table 4) as well as other studies (Reynolds et al., 2000; Naruoka et al., 2011) indicate that plasticity in terms of fertile Sp/P or per area is a very important attribute for yield progress. Free-tillering lines are better able to respond to the environment, thus shaping yield components in accordance with dynamic environmental cues such as precipitation, temperature, nutrient availability and plant competition (Assuero and Tognetti, 2010; Evers and Vos, 2013; Moeller et al., 2014). Therefore, higher tillering capacity may be beneficial in semi-arid Mediterranean-type environments, particularly under the projected increasingly erratic climate conditions (Wheeler and Von Braun, 2013).

### Prospects for wheat improvement

Empirical selection has largely improved drought adaptation in wheat through earlier flowering, reduced plant height and increased HI (Richards et al., 2010). Once the major genes for these traits have been fixed in the modern germplasm and exploited, there is a crucial need to identify and deploy genes or alleles conferring genuine improvement of the plants' physiological capacity to tolerate drought stress (Curtis and Halford, 2014; Lopes et al., 2014). Harnessing QTL alleles from wild relatives is essential for enhancing drought tolerance and other important traits (Tuberosa and Salvi, 2006). Nevertheless, most mapping studies utilize domesticated materials, while there has been hardly any attempt to map QTLs originated from wild germplasm (e.g., Table S6 and references therein). Therefore, the current study provides a unique opportunity to identify and reintroduce genes or alleles that were “left behind” during crop domestication and breeding, thereby enriching the modern gene pool with essential allelic diversity.

Tuberosa and Salvi (2006) argued that a major pitfall of most QTL studies lies in the selection of parental lines based on differences in target traits, rather than on their overall agronomic value. High drought resistance and water-use efficiency are often associated with reduced yield potential (Blum, 2009), and therefore selection of parental lines based mainly on the former criteria can lead to a mapping population that is characterized by poor productivity. A different approach was employed in our previous study (Peleg et al., 2005), in which we identified seven wild emmer natural populations with the greatest potential for wheat improvement based on a combination of high productivity and low susceptibility. One of these populations, included acc. G18-16, the parental line of our mapping study (Peleg et al., 2009) and the donor of the current introgression study. The use of a high-yielding cultivar (Langdon, Figure 2A) as the domesticated parent possibly eliminated from our genetic map QTL alleles for yield potential that are already available in the domesticated gene pool (that could have been identified with a less productive domesticated parent), thus highlighting the most beneficial wild alleles. Finally, the QTLs targeted for introgression in this work were chosen specifically for improvement of production capacity particularly under drought and for their stability across water availabilities, a strategy that proved successful across the two experiments in this study.

The current introgression lines were developed based on our previous mapping study, in which a total of 152 recombinant inbred lines were genotyped by a set of 690 SSR and DArT markers (Peleg et al., 2009), yielding a relatively low resolution QTL map. As a result, relatively large genomic regions were targeted for introgression. These large chromosomal segments contain hundreds of genes, many of which may have negative effects on the performance of the resultant lines, on their own or in interaction with either other genes or environmental factors (Salvi and Tuberosa, 2015). It is possible that the NILs that failed to validate the QTL effects in all or specific backgrounds reflected such negative effects. Moreover, elite cultivars present an optimal balance between all plant systems and gene networks, which might have been interrupted by such large introgressions. Modern high-throughput genotyping (e.g., SNP marker chips) and phenotyping (e.g., remote sensing) technologies, which have recently become more readily available and affordable, enable the use of large plant populations with thousands of genetic markers, and are thus expected to improve map resolution and enable more focused introgression studies, paving the way to a better exploration of genetic resources.

The introgression of favorable traits from wild relatives was recognized as a potential pathway for wheat improvement long ago (Aaronsohn, 1910). Nevertheless, a recent genome-wide diversity study of landraces and modern cultivars of hexaploid wheat suggests that past efforts have not notably altered the genetic composition of elite cultivars (Cavanagh et al., 2013). It was also noted that the contribution of advanced genomics-assisted technologies to develop drought-resistant cultivars has been limited so far (Tuberosa and Salvi, 2006; Salvi and Tuberosa, 2015).

## Concluding remarks

The current study provides first evidence that introgression of ancestral QTL alleles from wild emmer wheat can enhance productivity and drought resistance in domesticated wheat. A number of NILs exhibited significant advantage over their recurrent parents under the test environment, i.e., drip irrigated small plots in a rain protected facility. However, under commercial open field conditions competition and genotype x environment interactions might affect plant performances. Preliminary results from an ongoing study suggest that selected NILs maintain their advantage over the parental genotypes also in larger field plots, though to a smaller extent. Other current studies in our lab deal with physiological mechanisms underlying drought responses in selected NILs and fine mapping of selected genomic regions. The novel results obtained thus far and the findings of our current studies may pave the way to improving wheat productivity in arid regions, thereby enhancing global food security under climate change toward increasing aridity.

## Author contributions

LM student, marker assisted selection for development of near isogenic lines, phenotyping, data analysis and interpretation, manuscript preparation. VB, marker assisted selection for development of near isogenic lines, phenotyping, manuscript review. TF co-principle investigator, coordination, and guidance of marker assisted selection for development of near isogenic lines, manuscript review. FO, SNP genotyping of near isogenic lines, manuscript review. GL marker assisted selection for development of near isogenic lines, manuscript review. TK co-principle investigator, coordination, and guidance of marker assisted selection for development of near isogenic lines, manuscript review. YS principle investigator, coordination, supervision and guidance of marker assisted selection for development of near isogenic lines, phenotyping, data analysis and interpretation, manuscript preparation.

### Conflict of interest statement

The authors declare that the research was conducted in the absence of any commercial or financial relationships that could be construed as a potential conflict of interest.

## Supplementary material

Figure S1 **Pedigree of the developed near-isogenic lines and near-isogenic controls**. G and P designate the wild and domesticated alleles at the target region. Genotypes indicated by strikeout line were excluded from the backcross program.

Table S1 **SNP genotyping of selected NILs, NICs and parental lines for chromosomes 7A and 2B, aligned with the high density tetraploid wheat consensus map (Maccaferri et al., 2015, Table S2)**. Yellow highlighted cells indicate the target genomic regions (columns A & R) and the estimated introgressed segments (columns M-P & AD-AG); markers used for selection or alternative co-located markers are highlighted in Red. (1) A, G, C, and T indicate the respective nucleotides; (2) G, L, P, and NP indicate wild donor G18-16, Langdon and recurrent parent alleles, or non-polymorphic SNP, respectively.

Table S2 **Analysis of variance for total dry matter (TotDM), grain yield (GY), their susceptibility (TotDM-s and GY-s), days from planting to heading (DP–H), spike number per plant (Sp/P), grain number per spike (G/Sp), 1000 grain weight (TGW), harvest index (HI), culm length (CL), osmotic potential (OP), and osmotic adjustment (OA) in the 2012–13 experiment (Year 1)**. In parentheses, degrees of freedom for S and OA values.

Table S3 **Analysis of variance for total dry matter (TotDM), grain yield (GY), their susceptibility (TotDM-s and GY-s), days from planting to heading (DP–H), spike number per plant (Sp/P), grain number per spike (G/Sp), 1000 grain weight (TGW), harvest index (HI), culm length (CL), osmotic potential (OP) and osmotic adjustment (OA) in the 2013–14 experiment (Year 2)**. In parentheses, degrees of freedom for S and OA values.

Table S4 **Spike per plant (Sp/P), grains per spike (G/Sp) and 1000 grain weight (TGW) under the well-watered (WW) and water-limited (WL) treatments in Year 1 and Year 2**.

Table S5 **Harvest index (HI), culm length (CL) and osmotic potential (OP) under the well-watered (WW) and water-limited (WL) treatments, and calculated osmotic adjustment (OA) in Year 1 and Year 2**.

Table S6 **Summary of published QTLs or genes for productivity and drought-related traits, mapped to chromosome 7AS in durum and bread wheat: GY, grain yield; Sp/P, spikes per plant; TGW, 1000 grain weight; DP–H, days from planting to heading; WW, well-watered; WL, water-limited**.

## Abbreviations

Chr.: chromosome
CL: culm length
DP–H: days from planting to heading
G/Sp: grain number per spike
GY: grain yield
HI: harvest index
NIC: near-isogenic control
NIL: near-isogenic line
OA: osmotic adjustment
OP: osmotic potential
PCA: principal component analysis
QTL: quantitative trait loci
S: drought susceptibility index
SNP: single nucleotide polymorphism
Sp/P: fertile spikes per plan
SSR: single sequence repeat
TGW: thousand grain weight
TotDM: total dry matter
WL: water-limited
WW: well-watered.
