# Supplementary figures and images for: Ancestral QTL Alleles from Wild Emmer Wheat Improve Drought Resistance and Productivity in Modern Wheat Cultivars
Source: Front Plant Sci. 2016 Apr 15;7:452. doi: 10.3389/fpls.2016.00452 (PMC4832586; doi:10.3389/fpls.2016.00452)

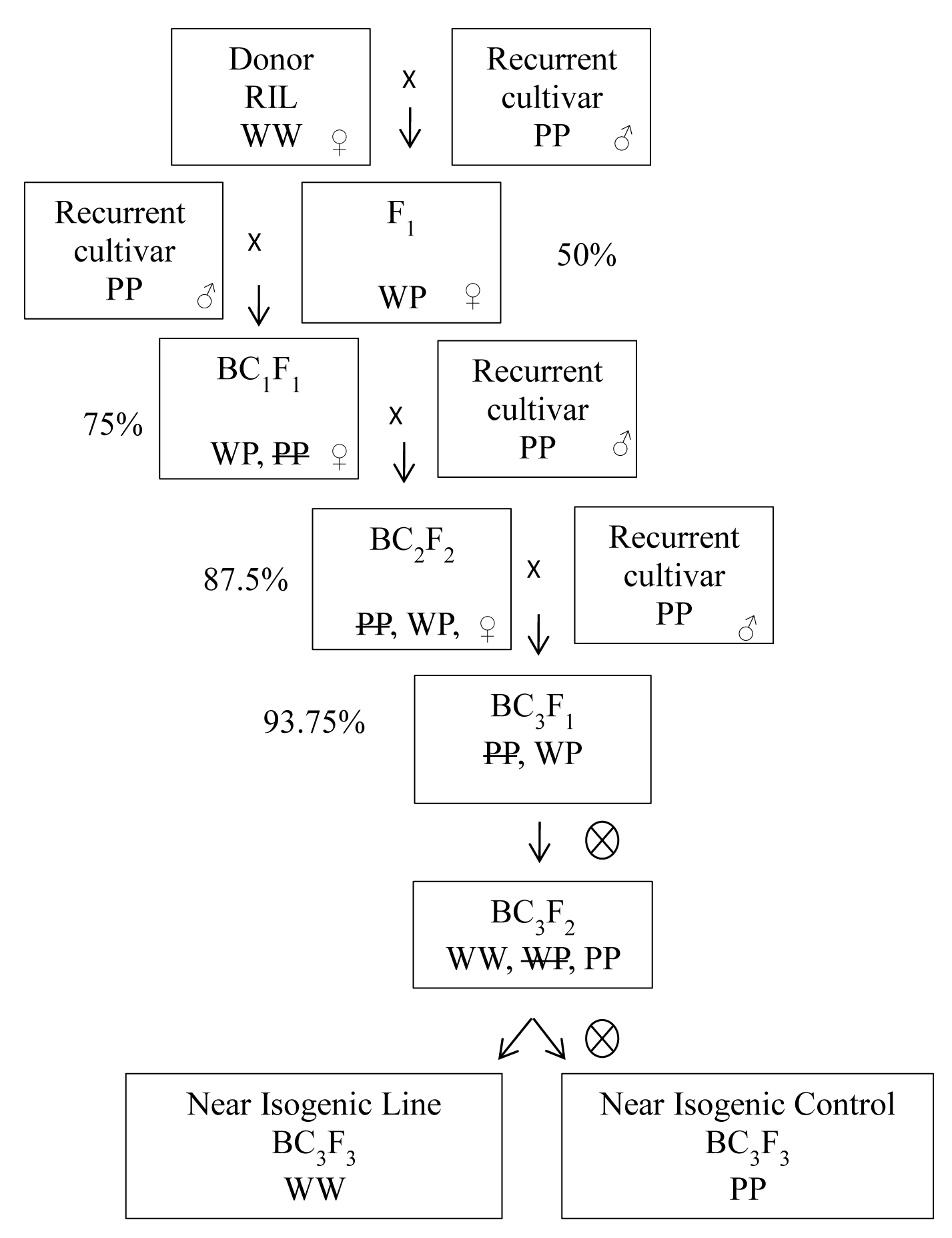

Supplement: Figure S1 — Pedigree of the developed near-isogenic lines and near-isogenic controls. G and P designate the wild and domesticated alleles at the target region. Genotypes indicated by strikeout line were excluded from the backcross program. [file Image1.JPEG]
