# Supplementary material for: Ancestral QTL Alleles from Wild Emmer Wheat Improve Drought Resistance and Productivity in Modern Wheat Cultivars
Source: Front Plant Sci. 2016 Apr 15;7:452. doi: 10.3389/fpls.2016.00452 (PMC4832586; doi:10.3389/fpls.2016.00452)
Supplement: Table S2 — Analysis of variance for total dry matter (TotDM), grain yield (GY), their susceptibility (TotDM-s and GY-s), days from planting to heading (DP–H), spike number per plant (Sp/P), grain number per spike (G/Sp), 1000 grain weight (TGW), harvest index (HI), culm length (CL), osmotic potential (OP), and osmotic adjustment (OA) in the 2012–13 experiment (Year 1). In parentheses, degrees of freedom for S and OA values. [file Table2.docx]

**Table S2** Analysis of variance for total dry matter (TotDM), grain yield (GY), their susceptibility (TotDM-s and GY-s), days from planting to heading (DP–H), spike number per plant (Sp/P), grain number per spike (G/Sp), thousand grain weight (TGW), harvest index (HI), culm length (CL), osmotic potential (OP) and osmotic adjustment (OA) in the 2012–13 experiment (Year 1). In parentheses, degrees of freedom for S and OA values.

| Source of | d.f. | F Ratio | | | | | | | | | | | | | | | | | | | | | | | | |  |
| --- | --- | --- | --- | --- | --- | --- | --- | --- | --- | --- | --- | --- | --- | --- | --- | --- | --- | --- | --- | --- | --- | --- | --- | --- | --- | --- | --- |
| variation |  | TotDM | | TotDM-S | | GY | | GY-s | | DP-H | | Sp/P | | G/Sp | | TGW | | HI | | CL | | OP | | OA | | |  |
| **Inbar and derivative lines** | | | |  |  |  |  |  |  |  |  |  |  |  |  |  |  |  |  |  |  |  |  |  | |  | |
| Genotype (G) | 12 (12) | 4.00 | ^***^ | 1.76 |  | 4.02 | ^***^ | 1.77 |  | 36.51 | ^***^ | 3.73 | ^***^ | 31.55 | ^***^ | 12.70 | ^***^ | 2.12 |  | 4.68 | ^***^ | 1.19 |  | 1.43 | |  | |
| Irrigation (I) | 1 | 31.69 | ^**^ |  |  | 25.71 | ^**^ |  |  | 0.88 |  | 27.50 | ^**^ | 2.70 |  | 28.39 | ^**^ | 3.98 |  | 27.44 | ^**^ | 15.00 |  |  | |  | |
| G x I | 12 | 1.76 |  |  |  | 1.89 | ^*^ |  |  | 1.78 | ^*^ | 1.16 |  | 1.61 | ^*^ | 2.62 | ^**^ | 0.77 |  | 1.13 |  | 0.88 |  |  | |  | |
| Block (B) | 4 (4) | 0.66 |  | 14.24 | ^***^ | 0.51 |  | 28.28 | ^***^ | 16.28 | ^**^ | 0.22 |  | 0.37 |  | 2.90 |  | 1.22 |  | 1.65 |  | 1.63 |  | 30.96 | | ^***^ | |
| Error a (B x I) | 4 | 7.94 | ^***^ |  |  | 11.39 | ^***^ |  |  | 1.16 |  | 11.81 | ^***^ | 9.67 | ^***^ | 3.03 | ^*^ | 26.89 |  | 8.37 | ^***^ | 18.75 | ^***^ | |  | |  |
| Error b (residual) | 113 (57) |  |  |  |  |  |  |  |  |  |  |  |  |  |  |  |  |  |  |  |  |  |  |  | |  | |
| **Uzan and derivative lines** | | |  |  |  |  |  |  |  |  |  |  |  |  |  |  |  |  |  |  |  |  |  |  | |  | |
| Genotype (G) | 3 (3) | 6.66 | ^***^ | 3.78 | ^*^ | 0.44 |  | 1.93 |  | 170.17 | ^***^ | 5.63 | ^**^ | 10.50 | ^***^ | 5.45 | ^**^ | 19.45 |  | 7.30 | ^***^ | 1.27 |  | 3.29 | | ^*^ | |
| Irrigation (I) | 1 | 21.15 | ^**^ |  |  | 14.62 | ^*^ |  |  | 7.13 | ^*^ | 28.45 | ^**^ | 4.65 |  | 2.71 |  | 3.09 |  | 23.06 | ^**^ | 20.20 | ^**^ |  | |  | |
| G x I | 3 | 1.22 |  |  |  | 0.45 |  |  |  | 0.38 |  | 2.05 |  | 0.64 |  | 3.96 | ^*^ | 0.53 |  | 1.70 |  | 1.96 |  |  | |  | |
| Block (B) | 4 (4) | 0.36 |  | 6.09 | ^**^ | 0.30 |  | 11.77 | ^***^ | 19.18 | ^**^ | 0.55 |  | 0.79 |  | 1.29 |  | 0.92 |  | 0.55 |  | 2.61 |  | 21.10 | | ^***^ | |
| Error a (B x I) | 4 | 4.49 | ^**^ |  |  | 4.85 | ^**^ |  |  | 0.37 |  | 3.61 | ^*^ | 3.71 | ^*^ | 1.88 |  | 8.05 |  | 8.17 | ^***^ | 7.59 | ^***^ | |  | |  |
| Error b (residual) | 41 (29) |  |  |  |  |  |  |  |  |  |  |  |  |  |  |  |  |  |  |  |  |  |  |  | |  | |
| **Bar-Nir and derivative lines** | | |  |  |  |  |  |  |  |  |  |  |  |  |  |  |  |  |  |  |  |  |  |  | |  | |
| Genotype (G) | 2 (2) | 1.76 |  | 2.80 |  | 3.98 | ^*^ | 5.01 | ^*^ | 30.92 | ^***^ | 1.20 |  | 0.62 |  | 23.46 | ^***^ | 0.75 |  | 57.70 | ^***^ | 0.94 |  | 2.51 | |  | |
| Irrigation (I) | 1 | 17.71 | ^***^ |  |  | 22.76 | ^**^ |  |  | 0.56 |  | 17.52 | ^**^ | 3.04 |  | 12.12 | ^*^ | 2.06 |  | 12.95 | ^*^ | 12.27 | ^*^ |  | |  | |
| G x I | 2 | 1.05 |  |  |  | 0.30 |  |  |  | 0.67 |  | 0.13 |  | 3.49 | ^*^ | 0.83 |  | 1.30 |  | 0.56 |  | 1.66 |  |  | |  | |
| Block (B) | 4 (4) | 0.32 |  | 6.56 |  | 0.47 |  | 10.40 | ^***^ | 4.24 |  | 0.12 |  | 1.00 |  | 1.62 |  | 1.16 |  | 1.22 |  | 1.67 |  | 15.19 | | ^***^ | |
| Error a (B x I) | 4 | 1.68 |  |  |  | 1.51 |  |  |  | 5.14 | ^**^ | 1.88 |  | 1.58 |  | 6.38 | ^***^ | 1.40 |  | 1.42 |  | 8.46 | ^***^ | |  | |  |
| Error b (residual) | 34 (16) |  |  |  |  |  |  |  |  |  |  |  |  |  |  |  |  |  |  |  |  |  |  |  | |  | |
| **Zahir and derivative lines** | | |  |  |  |  |  |  |  |  |  |  |  |  |  |  |  |  |  |  |  |  |  |  | |  | |
| Genotype (G) | 3 (3) | 8.36 | ^***^ | 0.32 |  | 8.12 | ^***^ | 1.06 |  | 22.22 | ^***^ | 4.83 | ^**^ | 9.16 | ^***^ | 0.37 |  | 5.77 | ^**^ | 49.48 | ^***^ | 0.17 |  | 0.47 | |  | |
| Irrigation (I) | 1 | 15.13 | ^*^ |  |  | 11.67 | ^*^ |  |  | 0.02 |  | 13.51 | ^*^ | 0.29 |  | 4.79 |  | 3.29 |  | 11.96 | ^*^ | 8.07 |  |  | | *** | |
| G x I | 2 | 0.11 |  |  |  | 0.27 |  |  |  | 3.43 | ^*^ | 0.72 |  | 1.04 |  | 0.45 |  | 3.89 | ^*^ | 0.49 |  | 0.31 |  |  | |  | |
| Block (B) | 4 (4) | 0.22 |  | 3.32 | ^*^ | 0.10 |  | 3.86 | ^*^ | 1.52 |  | 0.04 |  | 1.28 |  | 1.73 |  | 0.84 |  | 3.13 |  | 1.51 |  | 11.38^***^ | |  | |
| Error a (B x I) | 4 | 2.36 |  |  |  | 3.46 | ^*^ |  |  | 3.19 | ^*^ | 7.20 | ^***^ | 1.51 |  | 3.49 | ^*^ | 3.93 | ^*^ | 0.73 |  | 6.12 |  | |  | |  |
| Error b (residual) | 32 (21) |  |  |  |  |  |  |  |  |  |  |  |  |  |  |  |  |  |  |  |  |  |  |  | |  | |

*, **, *** *P* < 0.05, 0.01 and 0.001, respectively.
