# Supplementary material for: Ancestral QTL Alleles from Wild Emmer Wheat Improve Drought Resistance and Productivity in Modern Wheat Cultivars
Source: Front Plant Sci. 2016 Apr 15;7:452. doi: 10.3389/fpls.2016.00452 (PMC4832586; doi:10.3389/fpls.2016.00452)
Supplement: Table S3 — Analysis of variance for total dry matter (TotDM), grain yield (GY), their susceptibility (TotDM-s and GY-s), days from planting to heading (DP–H), spike number per plant (Sp/P), grain number per spike (G/Sp), 1000 grain weight (TGW), harvest index (HI), culm length (CL), osmotic potential (OP) and osmotic adjustment (OA) in the 2013–14 experiment (Year 2). In parentheses, degrees of freedom for S and OA values. [file Table3.DOCX]

**Table S3.**  Analysis of variance for total dry matter (TotDM), grain yield (GY), their susceptibility (TotDM-s and GY-s), days from planting to heading (DP–H), spike number per plant (Sp/P), grain number per spike (G/Sp), thousand grain weight (TGW), harvest index (HI), culm length (CL), osmotic potential (OP) and osmotic adjustment (OA) in the 2013–14 experiment (Year 2). In parentheses, degrees of freedom for S and OA values.

| Source of | d.f. | F Ratio |  |  |  |  |  |  |  |  |  |  | | | | | | | | | | | | | |
| --- | --- | --- | --- | --- | --- | --- | --- | --- | --- | --- | --- | --- | --- | --- | --- | --- | --- | --- | --- | --- | --- | --- | --- | --- | --- |
| variation |  | TotDM | | TotDM-s | | GY | | GY-s | | DP-H | | Sp/P | | G/Sp | | TGW | | HI | | CL | | OP | | OA | |
| **Inbar and derivative lines** | | |  |  |  |  |  |  |  |  |  |  |  |  |  |  |  |  |  |  |  |  |  |  |  |
| Genotype (G) | 12 (12) | 3.11 | ^***^ | 1.31 |  | 5.60 | ^***^ | 1.34 |  | 28.73 | ^***^ | 3.11 | ^***^ | 6.69 | ^***^ | 5.95 | ^***^ | 2.79 | ^**^ | 3.44 | ^***^ | 1.93 | ^*^ | 2.58 | ^**^ |
| Irrigation (I) | 1 | 88.01 | ^***^ |  |  | 75.58 | ^**^ |  |  | 2.81 |  | 56.27 | ^**^ | 30.61 | ^**^ | 0.67 |  | 0.22 |  | 170.17 | ^***^ | 19.24 | ^*^ |  |  |
| G x I | 12 | 1.14 |  |  |  | 1.33 |  |  |  | 1.39 |  | 1.28 |  | 1.21 |  | 1.89 | ^*^ | 2.09 | ^*^ | 1.12 |  | 1.32 |  |  |  |
| Block (B) | 4 (4) | 1.24 |  | 1.99 |  | 0.70 |  | 1.57 |  | 1.43 |  | 1.30 |  | 4.14 |  | 8.07 | ^*^ | 10.25 | ^*^ | 2.20 |  | 1.81 |  | 13.27 | ^***^ |
| Error a (B x I) | 4 | 1.40 |  |  |  | 2.57 |  |  |  | 21.21 | ^***^ | 2.67 | ^*^ | 0.83 |  | 0.68 |  | 0.26 |  | 0.48 |  | 7.33 | ^***^ |  |  |
| Error b (residual) | 102 (49) |  |  |  |  |  |  |  |  |  |  |  |  |  |  |  |  |  |  |  |  |  |  |  |  |
| **Uzan and derivative lines** | | |  |  |  |  |  |  |  |  |  |  |  |  |  |  |  |  |  |  |  |  |  |  |  |
| Genotype (G) | 8 (8) | 7.17 | ^***^ | 2.06 |  | 4.96 | ^***^ | 3.78 | ^**^ | 48.62 | ^***^ | 7.56 | ^***^ | 4.62 | ^***^ | 10.00 | ^***^ | 1.56 |  | 11.96 | ^***^ | 1.12 |  | 2.62 | ^*^ |
| Irrigation (I) | 1 | 57.03 | ^**^ |  |  | 45.38 | ^**^ |  |  | 0.23 |  | 188.11 | ^***^ | 4.56 |  | 7.28 | ^*^ | 0.02 |  | 69.45 | ^***^ | 13.21 |  |  |  |
| G x I | 8 | 1.54 |  |  |  | 2.14 | ^*^ |  |  | 1.38 |  | 2.37 | ^*^ | 1.49 |  | 2.32 | ^*^ | 2.32 | ^*^ | 0.83 |  | 1.67 |  |  |  |
| Block (B) | 4 (4) | 1.00 |  | 0.99 |  | 0.10 |  | 0.61 |  | 0.69 |  | 4.95 |  | 1.90 |  | 6.98 | ^*^ | 2.45 |  | 0.15 |  | 1.92 |  | 5.03 | ^**^ |
| Error a (B x I) | 4 | 1.35 |  |  |  | 1.20 |  |  |  | 11.17 | ^***^ | 0.39 |  | 2.11 |  | 0.63 |  | 1.18 |  | 1.42 |  | 4.74 | ^**^ |  |  |
| Error b (residual) | 70 (35) |  |  |  |  |  |  |  |  |  |  |  |  |  |  |  |  |  |  |  |  |  |  |  |  |
| **Bar-Nir and derivative lines** | | |  |  |  |  |  |  |  |  |  |  |  |  |  |  |  |  |  |  |  |  |  |  |  |
| Genotype (G) | 4 (4) | 0.95 |  | 3.10 | ^*^ | 2.09 |  | 3.69 | ^*^ | 25.57 | ^***^ | 2.04 |  | 1.08 |  | 17.29 | ^***^ | 11.43 | ^***^ | 34.73 | ^***^ | 2.99 | ^*^ | 9.02 | ^***^ |
| Irrigation (I) | 1 | 70.69 | ^***^ |  |  | 53.55 | ^***^ |  |  | 3.51 |  | 132.39 | ^***^ | 8.96 | ^*^ | 2.94 |  | 0.08 |  | 13.52 | ^*^ | 3.86 |  |  |  |
| G x I | 4 | 2.15 |  |  |  | 1.76 |  |  |  | 0.44 |  | 4.80 | ^**^ | 0.78 |  | 1.41 |  | 2.49 |  | 0.59 |  | 1.78 |  |  |  |
| Block (B) | 4 (4) | 1.34 |  | 0.52 |  | 0.89 |  | 1.43 |  | 6.50 | ^*^ | 2.44 |  | 2.78 |  | 2.00 |  | 1.34 |  | 0.97 |  | 0.90 |  | 12.94 | ^***^ |
| Error a (B x I) | 4 | 0.74 |  |  |  | 0.72 |  |  |  | 5.97 | ^***^ | 0.60 |  | 1.28 |  | 2.89 | ^*^ | 2.06 |  | 2.14 |  | 9.11 | ^***^ |  |  |
| Error b (residual) | 53 (27) |  |  |  |  |  |  |  |  |  |  |  |  |  |  |  |  |  |  |  |  |  |  |  |  |
| **Zahir and derivative lines** | | |  |  |  |  |  |  |  |  |  |  |  |  |  |  |  |  |  |  |  |  |  |  |  |
| Genotype (G) | 3 (3) | 6.73 | ^**^ | 0.46 |  | 5.94 | ^**^ | 0.27 |  | 28.94 | ^***^ | 5.01 | ^*^ | 10.53 | ^***^ | 1.01 |  | 1.66 |  | 42.63 | ^***^ | 1.72 |  | 1.42 |  |
| Irrigation (I) | 1 | 36.97 | ^**^ |  |  | 58.49 | ^**^ |  |  | 2.04 |  | 40.45 | ^**^ | 20.61 | ^*^ | 0.02 |  | 0.24 |  | 6.55 |  | 3.63 |  |  |  |
| G x I | 3 | 0.38 |  |  |  | 0.28 |  |  |  | 0.67 |  | 0.36 |  | 1.56 |  | 1.99 |  | 1.83 |  | 3.01 | ^*^ | 0.74 |  |  |  |
| Block (B) | 4 (4) | 0.32 |  | 0.60 |  | 0.27 |  | 0.50 |  | 2.93 |  | 1.07 |  | 0.12 |  | 3.41 |  | 1.42 |  | 0.73 |  | 5.23 |  | 1.20 |  |
| Error a (B x I) | 4 | 1.78 |  |  |  | 0.91 |  |  |  | 4.22 | ^*^ | 1.18 |  | 1.17 |  | 1.07 |  | 1.46 |  | 2.54 |  | 0.72 |  |  |  |
| Error b (residual) | 46 (22) |  |  |  |  |  |  |  |  |  |  |  |  |  |  |  |  |  |  |  |  |  |  |  |  |

*, **, *** *P* < 0.05, 0.01 and 0.001, respectively.
