# Supplementary material for: Ancestral QTL Alleles from Wild Emmer Wheat Improve Drought Resistance and Productivity in Modern Wheat Cultivars
Source: Front Plant Sci. 2016 Apr 15;7:452. doi: 10.3389/fpls.2016.00452 (PMC4832586; doi:10.3389/fpls.2016.00452)
Supplement: Table S4 — Spike per plant (Sp/P), grains per spike (G/Sp) and 1000 grain weight (TGW) under the well-watered (WW) and water-limited (WL) treatments in Year 1 and Year 2. [file Table4.DOCX]

**Table S4.** Spike per plant (Sp/P), grains per spike (G/Sp) and thousand grain weight (TGW) under the well-watered (WW) and water-limited (WL)

| **Chro-mosome** | **Genotype** | | | | **SSR** | | **Sp/P** | | | | **G/Sp** | | | | **TGW (g)** | | | |
| --- | --- | --- | --- | --- | --- | --- | --- | --- | --- | --- | --- | --- | --- | --- | --- | --- | --- | --- |
|  |  | | | **allele** | | | **Year 1** | | **Year 2** | | **Year 1** | | **Year 2** | | **Year 1** | | **Year 2** | |
|  |  | | | | |  | **WW** | **WL** | **WW** | **WL** | **WW** | **WL** | **WW** | **WL** | **WW** | **WL** | **WW** | **WL** |
|  | | **G18-16** | | | | | 14.3 | 9.0 | 12.2 | 4.9 | 25.2 | 19.2 | 56.6 | 47.1 | 42.5 | 49.7 | 36.5 | 33.8 |
|  | | | **Langdon** | | | | 12.1 | 7.0 | 10.1 | 6.1 | 61.4 | 41.2 | 72.3 | 54.6 | 51.2 | 55.0 | 44.6 | 39.5 |
| **Recurrent Parent -Bread cv. Bar Nir** | | | | | | | 12.7 | 7.4 | 9.6 | 4.8 | 56.8 | 59.3 | 53.7 | 44.1 | 49.1 | 41.2 | 47.6 | 47.5 |
| **Chr.7AS** | NIL-B-7A-1 | | | | | G--G | 12.5 | 7.6 | 8.8 | 6.3 | 60.7 | 58.9 | 52.9 | 40.7 | 57.5*** | 48.1** | 53.3***ccc | 55.4***cc |
|  | NIL-B-7A-2 | | | | | G--G | 13.5 | 9.3 | 8.3 | 6.8 | 55.2 | 63.3 | 48.2 | 45.7 | 54.8** | 47.3** | 48.3 | 50.0 |
|  | NIL-B-7A-3 | | | | | P--G |  |  | 11.6 | 5.8 |  |  | 51.6 | 47.4 |  |  | 50.0 | 51.5** |
|  | NIC-B-7A-2 | | | | | P--P |  |  | 7.5 | 4.8 |  |  | 64.7 | 50.2 |  |  | 48.8 | 47.8 |
| **Recurrent Parent - Bread cv.Zahir** | | | | | | | 7.8 | 5.2 | 6.0 | 3.7 | 62.6 | 61.3 | 66.3 | 51.6 | 54.5 | 49.1 | 52.3 | 55.2 |
| **Chr.7AS** | NIL-Z-7A-2 | | | | | G--P | 8.5 | 4.4 | 6.9 | 3.4 | 51.1** | 58.1 | 47.4*** | 42.6* | 55.6 | 50.0 | 55.8_0.05_ | 52.2 |
|  | NIL-Z-7A-5 | | | | | G--P | 9.6_0.07_ | 6.5* | 7.9* | 5.3* | 66.4 | 69.4_0.06_ | 62.1 | 48.9 | 54.9 | 49.7 | 56.01* | 55.49 |
|  | NIL-Z-7A-4 | | | | | P--G | 8.4 | 4.8 | 7.9* | 5.2_0.05_ | 53.8* | 50.5* | 51.2*** | 43.3_0.07_ | 54.4 | 51.9 | 52.9 | 54.4 |
| **Recurrent Parent - Durum cv. Inbar** | | | | | | | 9.2 | 4.5 | 7.0 | 4.8 | 59.6 | 52.7 | 64.6 | 47.5 | 69.8 | 67.3 | 63.1 | 64.9 |
| **Chr.1BL** | NIL-I-1B-1 | | | | | G-G-P | 9.8 | 4.5 | 6.8 | 4.0 | 42.3*** | 38.1*** | 37.8*** | 36.8* | 72.5cc | 63.1*ccc | 75.1***c | 70.6* |
|  | NIL-I-1B-2 | | | | | G-G-P | 7.9 | 5.4 | 9.1* | 4.6 | 39.5*** | 41.4*** | 37.6*** | 39.7 | 71.6cc | 69.3 | 71.7** | 66.1 |
|  | NIC-I-1B-1,2 | | | | | P-P-P | 10.1 | 6.3* | 8.2 | 5.2 | 41.4*** | 37.3*** | 46.9*** | 39.5 | 78.8*** | 70.6* | 68.8* | 71.2* |
| **Chr.2BS** | NIL-I-2B-1 | | | | | G-G-G | 11.24* | 6.4* | 6.6 | 4.8 | 48.6*** | 43.2*** | 44.0*** | 42.7 | 66.9 | 64.1 | 60.5 | 66.3 |
|  | NIL-I-2B-2 | | | | | G-G-G | 10.9* | 4.8 | 6.3 | 4.2 | 58.3 | 46.0** | 48.4** | 41.2 | 69.1 | 64.1 | 62.2 | 65.2 |
|  | NIL-I-2B-3 | | | | | G-P-P | 7.7 | 4.6 | 6.9 | 4.7 | 66.2 | 56.5 | 60.6 | 49.5 | 62.4*** | 61.2*** | 58.3 | 64.3 |
| **Chr.7AS** | NIL-I-7A-1 | | | | | G--G | 11.8** | 5.4 | 9.1* | 5.1 | 50.5***ccc | 46.5* | 46.8***c | 43.8 | 76.3***cc | 74.0***c | 69.0* | 75.5***ccc |
|  | NIL-I-7A-2 | | | | | G--G | 9.7 | 5.0 | 7.5cc | 5.5 | 49.7***cc | 45.14** | 52.4* | 42.3 | 72.2 | 66.0 | 69.0* | 68.3 |
|  | NIC-I-7A-1 | | | | | P--P | 12.4*** | 6.1* | 10.2*** | 6.2 | 64.9 | 54.4* | 59.1 | 52.3 | 69.6 | 68.8 | 66.5 | 64.3 |
| **Chr.7BS** | NIL-I-7B-1 | | | | | G--G | 10.5 | 5.8 | 9.3* | 4.4 | 41.7*** | 41.6*** | 45.5*** | 38.10.05 | 72.9 | 65.3 | 67.0 | 65.0c |
|  | NIL-I-7B-2 | | | | | G--G | 9.4 | 5.9 | 9.1* | 5.0 | 41.2*** | 43.7*** | 48.5** | 34.8** | 75.6*** | 66.1 | 70.0* | 65.6 |
|  | NIC-I-7B-1,2 | | | | | P--P | 11.2 | 5.9 | 8.3 | 4.6 | 41.3*** | 37.9*** | 45.2 | 35.9 | 76.3*** | 68.6 | 70.3* | 71.3* |
| **Recurrent Parent - Durum cv. Uzan** | | | | | | | 8.4 | 4.3 | 5.2 | 3.3 | 55.7 | 50.6 | 54.8 | 40.2 | 65.9 | 58.0 | 62.3 | 59.6 |
| **Chr.1BL** | NIL-U-1B-1 | | | | | G-G-G | 9.7 | 4.1 | 6.1 | 5.7** | 51.3 | 46.1 | 50.1 | 48.8 | 69.1 | 66.5*** | 63.2 | 60.7 |
|  | NIL-U-1B-2 | | | | | G-G-G | 7.8 | 5.3 | 7.8** | 3.6 | 45.7*** | 43.7** | 54.4 | 42.9 | 67.5 | 62.5* | 65.7 | 57.8 |
|  | NIL-U-1B-3 | | | | | G-G-G |  |  | 6.7 | 3.6 |  |  | 50.6 | 45.3 |  |  | 66.4 | 63.8* |
|  | NIL-U-1B-4 | | | | | G-G-P |  |  | 5.7 | 4.7 |  |  | 50.0 | 52.7* |  |  | 64.5 | 65.6** |
| **Chr.2BS** | NIL-U-2B-1 | | | | | G-G-G | 11.5** | 6.6* | 10.2*** | 6.67*** | 55.5 | 48.3 | 64.6 | 65.7*** | 59.6* | 65.1* | 57.7 | 48.7*** |
|  | NIL-U-2B-2 | | | | | P-G-G |  |  | 9.1*** | 4.6 |  |  | 51.2 | 42.5 |  |  | 57.7 | 62.6 |
|  | NIL-U-2B-3 | | | | | P-P-G |  |  | 8.3*** | 4.92 |  |  | 46.4 | 44.9 |  |  | 67.6*c | 66.3** |
|  | NIC-U-2B-3 | | | | | P-P-P |  |  | 7.0 | 4.9 |  |  | 52.5 | 42.6 |  |  | 62.1 | 62.9 |

treatments in Year 1 and Year 2

Mean comparisons by t-test between each of the lines and its recurrent parents (*, **, ***) or its near isogenic control (NIC, c, cc, ccc) under a specific irrigation treatment at *P* < 0.05, 0.01 and 0.001, respectively. SSR alleles: G-wild emmer wheat; P- parental cultivar.
