# Supplementary material for: Ancestral QTL Alleles from Wild Emmer Wheat Improve Drought Resistance and Productivity in Modern Wheat Cultivars
Source: Front Plant Sci. 2016 Apr 15;7:452. doi: 10.3389/fpls.2016.00452 (PMC4832586; doi:10.3389/fpls.2016.00452)
Supplement: Table S5 — Harvest index (HI), culm length (CL) and osmotic potential (OP) under the well-watered (WW) and water-limited (WL) treatments, and calculated osmotic adjustment (OA) in Year 1 and Year 2. [file Table5.DOCX]

**Table S5.** Harvest index (HI), culm length (CL) and osmotic potential (OP) under the well-watered (WW) and water-limited (WL) treatments, and calculated osmotic adjustment (OA) in Year 1 and Year 2.

| **Chro-mosom** | **Genotype** | **SSR**  **alleles** | **HI** | | | | **CL (cm)** | | | | **OP (Mpa)** | | | | | |
| --- | --- | --- | --- | --- | --- | --- | --- | --- | --- | --- | --- | --- | --- | --- | --- | --- |
|  |  |  | **Year 1** | | **Year 2** | | **Year 1** | | **Year 2** | | **Year 1** | | | **Year 2** | | |
|  |  |  | **WW** | **WL** | **WW** | **WL** | **WW** | **WL** | **WW** | **WL** | **WW** | **WL** | **OA** | **WW** | **WL** | **OA** |
|  | **G18-16** |  | 0.23 | 0.22 | 0.18 | 0.18 | 103 | 65 | 93 | 66 | -1.17 | -1.47 | 0.30 | -1.02 | -1.36 | 0.34 |
|  | **Langdon** |  | 0.30 | 0.23 | 0.31 | 0.29 | 161 | 118 | 175 | 129 | -1.43 | -1.46 | 0.03 | -1.05 | -1.14 | 0.10 |
| **Recurrent Parent -Bread cv. Bar Nir** | | | 0.57 | 0.52 | 0.52 | 0.55 | 60 | 56 | 59 | 50 | -1.32 | -1.76 | 0.44 | -1.19 | -1.33 | 0.14 |
| **Chr.7AS** | NIL-B-7A-1 | G--G | 0.57 | 0.56 | 0.60*ccc | 0.51c | 71*** | 64*** | 68**c | 61***cc | -1.35 | -1.64 | 0.28 | -1.25 | -1.37 | 0.12cc |
|  | NIL-B-7A-2 | G--G | 0.56 | 0.56 | 0.56ccc | 0.53c | 75*** | 70*** | 70*** | 62***ccc | -1.26 | -1.61 | 0.38 | -1.26 | -1.52***c | 0.25*ccc |
|  | NIL-B-7A-3 | P--G |  |  | 0.53ccc | 0.58cc |  |  | 57ccc | 51ccc |  |  |  | -1.19 | -1.42 | 0.23ccc |
|  | NIC-B-7A-2 | P--P |  |  | 0.35*** | 0.43** |  |  | 76*** | 73*** |  |  |  | -1.42 | -1.38 | 0.04*** |
| **Recurrent Parent - Bread cv.Zahir** | | | 0.56 | 0.54 | 0.53 | 0.57 | 74 | 69 | 68 | 63 | -1.17 | -1.46 | 0.30 | -1.12 | -1.27 | 0.17 |
| **Chr.7AS** | NIL-Z-7A-2 | G--P | 0.54 | 0.46*** | 0.61* | 0.54 | 66* | 66 | 64 _0.05_ | 58 _0.09_ | -1.16 | -1.56 | 0.40 | -1.12 | -1.23 | 0.10 |
|  | NIL-Z-7A-5 | G--P | 0.55 | 0.51 | 0.51 | 0.53 | 83** | 75* | 76*** | 67* | -1.18 | -1.54 | 0.30 | -1.15 | -1.22 | 0.07 |
|  | NIL-Z-7A-4 | P--G | 0.57 | 0.56 | 0.55 | 0.52 | 56*** | 51*** | 52*** | 53** | -1.17 | -1.48 | 0.31 | -1.32* | -1.29 | 0.03* |
| **Recurrent Parent - Durum cv. Inbar** | | | 0.52 | 0.45 | 0.51 | 0.53 | 77 | 66 | 70 | 58 | -1.26 | -1.63 | 0.37 | -1.07 | -1.27 | 0.20 |
| **Chr.1BL** | NIL-I-1B-1 | G-G-P | 0.54 | 0.48 | 0.52 | 0.50 | 72**cc | 64cc | 68c | 61 | -1.12* | -1.56 | 0.44 | -1.09 | -1.31 | 0.22c |
|  | NIL-I-1B-2 | G-G-P | 0.55 | 0.52 | 0.50 | 0.52 | 73c | 66c | 69c | 64* | -1.16 | -1.57 | 0.47 | -1.06c | -1.26 | 0.20c |
|  | NIC-I-1B-1,2 | P-P-P | 0.51 | 0.47 | 0.50 | 0.53 | 80 | 70* | 77* | 66* | -1.23 | -1.54 | 0.30 | -1.20* | -1.26 | 0.06 |
| **Chr.2BS** | NIL-I-2B-1 | G-G-G | 0.52 | 0.47 | 0.47 | 0.49 | 76 | 68 | 65 | 63 | -1.22 | -1.60 | 0.38 | -1.13 | -1.45** | 0.33* |
|  | NIL-I-2B-2 | G-G-G | 0.52 | 0.42 | 0.50 | 0.46* | 76 | 63 | 62** | 56 | -1.31 | -1.66 | 0.35 | -1.12 | -1.41* | 0.29 |
|  | NIL-I-2B-3 | G-P-P | 0.53 | 0.44 | 0.47 | 0.47*** | 76 | 66 | 66 | 60 | -1.35 | -1.54 | 0.19* | -1.08 | -1.42** | 0.35* |
| **Chr.7AS** | NIL-I-7A-1 | G--G | 0.54 | 0.48 | 0.51 | 0.49 | 7 | 63cc | 67c | 61 | -1.29 | -1.51 | 0.22 | -1.09 | -1.25 | 0.15 |
|  | NIL-I-7A-2 | G--G | 0.56 | 0.47 | 0.50 | 0.53 | 72cc | 62*ccc | 74 | 67 | -1.21 | -1.58 | 0.37 | -1.10 | -1.24 | 0.14 |
|  | NIC-I-7A-1 | P--P | 0.53 | 0.48 | 0.50 | 0.52 | 79 | 70* | 74 | 63 | -1.19 | -1.61 | 0.43 | -1.04 | -1.30 | 0.26 |
| **Chr.7BS** | NIL-I-7B-1 | G--G | 0.49 | 0.47 | 0.48 | 0.46** | 70*** | 66 | 69 | 58 | -1.17 | -1.50 | 0.33 | -1.11 | -1.38* | 0.27 |
|  | NIL-I-7B-2 | G--G | 0.52 | 0.46 | 0.48 | 0.52cc | 73* | 67 | 72 | 62 | -1.17 | -1.58 | 0.41 | -1.06 | -1.28 | 0.22 |
|  | NIC-I-7B-1,2 | P--P | 0.51 | 0.42 | 0.46* | 0.45** | 76 | 66 | 73 | 62 | -1.20 | -1.58 | 0.38 | -1.01 | -1.24 | 0.23 |
| **Recurrent Parent - Durum cv. Uzan** | | | 0.54 | 0.48 | 0.52 | 0.45 | 73 | 61 | 65 | 54 | -1.27 | -1.70 | 0.43 | -1.18 | -1.40 | 0.23 |
| **Chr.1BL** | NIL-U-1B-1 | G-G-G | 0.52 | 0.46 | 0.39** | 0.48 | 79** | 65 | 69 | 60 | -1.31 | -1.56* | 0.25** | -1.26 | -1.32 | 0.07* |
|  | NIL-U-1B-2 | G-G-G | 0.50 | 0.43* | 0.49 | 0.44 | 76 | 58 | 71 | 54 | -1.27 | -1.74 | 0.46 | -1.15 | -1.45 | 0.31 |
|  | NIL-U-1B-3 | G-G-G |  |  | 0.48 | 0.44 |  |  | 66 | 57 |  |  |  | -1.12 | -1.39 | 0.27 |
|  | NIL-U-1B-4 | G-G-P |  |  | 0.35*** | 0.49 |  |  | 66 | 60* |  |  |  | -1.19 | -1.36 | 0.17 |
| **Chr.2BS** | NIL-U-2B-1 | G-G-G | 0.39*** | 0.38*** | 0.42* | 0.40 | 69 | 58 | 84*** | 73*** | -1.20 | -1.53 | 0.29 | -1.17 | -1.19*** | 0.03**c |
|  | NIL-U-2B-2 | P-G-G |  |  | 0.45 | 0.42 |  |  | 70 | 60 |  |  |  | -1.18 | -1.36 | 0.18 |
|  | NIL-U-2B-3 | P-P-G |  |  | 0.49c | 0.52 |  |  | 72* | 60* |  |  |  | -1.20 | -1.40 | 0.20 |
|  | NIC-U-2B-3 | P-P-P |  |  | 0.45 | 0.41 |  |  | 66 | 55 |  |  |  | -1.18 | -1.38 | 0.20 |

Mean comparisons by t-test between each of the lines and its recurrent parents (*, **, ***) or its near isogenic control (NIC, c, cc, ccc) under a specific irrigation treatment at *P* < 0.05, 0.01 and 0.001, respectively. SSR alleles: G-wild emmer wheat; P-parental cultivar.
