# Supplementary material for: Ancestral QTL Alleles from Wild Emmer Wheat Improve Drought Resistance and Productivity in Modern Wheat Cultivars
Source: Front Plant Sci. 2016 Apr 15;7:452. doi: 10.3389/fpls.2016.00452 (PMC4832586; doi:10.3389/fpls.2016.00452)
Supplement: Table S6 — Summary of published QTLs or genes for productivity and drought-related traits, mapped to chromosome 7AS in durum and bread wheat: GY, grain yield; Sp/P, spikes per plant; TGW, 1000 grain weight; DP–H, days from planting to heading; WW, well-watered; WL, water-limited. [file Table6.DOCX]

**Table S6.** Summary of published QTLs or genes for productivity and drought-related traits, mapped to chromosome 7AS in durum and bread wheat: GY, grain yield; Sp/P, spikes per plant; TGW, thousand grain weight; DP–H, days from planting to heading; WW, well-watered; WL, water-limited.

| **Plant Material, Environment** | **Mapped traits** | | | | | **Reference** |
| --- | --- | --- | --- | --- | --- | --- |
|  | **GY** | **Sp/P** | **TGW** | **DP-H** | **Other traits** |  |
| **Durum wheat:** |  |  |  |  |  |  |
| RIL (Kofa x Svevo), 6 env |  |  |  |  | Culm length | Maccaferri *et al.,* 2008 |
| RIL (Langdon x wild emmer acc. G18-16), WW & WL | + |  |  |  | Total DM, flag-leaf rolling & length | Peleg *et al.,* 2009b |
| F_2_ (Aiganfanmai x Langdon), WW |  |  |  |  | Culm length | Peng *et al.,* 2011 |
| 189 elite durum wheat accessions |  |  |  | + |  | Maccaferri *et al.,* 2011 |
| **Bread wheat:** |  |  |  |  |  |  |
| RILs (Songlen x Condor*4/3Ag14), WW |  |  |  |  | Osmoregulation (K accumulation) | Morgan and Tan, 1996 |
| BC2F3 (Prinz x W-7984), 4 env |  | + | + |  |  | Huang et al., 2003 |
| RIL (Rye Selection111 × Chinese Spring), 6 env |  |  | + |  |  | Kumar *et al.,* 2006 |
| DH (Chinese Spring × SQ1), 13 env | + | + | + |  |  | Quarrie *et al.,* 2006 |
| RILs (WL711 x PH132 & Opata85 x W7984), 6 env | + | + |  |  |  | Kumar *et al.,* 2007 |
| DH population (Hanxuan x Lumai 14), WW & WL |  |  |  |  | Remobilization efficiency of stem carbohydrates | Yang *et al.,* 2007 |
| DHs (Cranbrook x Halberd, Sunco x Tasman, CD87 x Katepwa), 5 env |  |  |  |  | Carbon isotope discrimination | Rebetzke *et al.,* 2008 |
| DHs (Avalon x Cadenza, Charger x Badger, Spark x Rialto, Savannah x Rialto), 13 env |  |  |  | + |  | Griffiths *et al.,* 2009 |
| RIL (Seri x Babax), WL & heat |  |  |  |  | Canopy temperature | Pinto *et al.,* 2010 |
| DHs (Cranbrook × Halberd & Excalibur × Kukri),  hydroponics & field |  |  |  |  | Na^+^ exclusion | Shavrukov *et al.,* 2011 |
| DH (RAC875 x Kukri), heat, WW & WL | + |  | + | + | Glaucousness, G/m^2^, NDVI | Bennett *et al.,* 2012 |
| DH (Opata x SH223), WW & WL |  |  |  |  | Chlorophyll content | Ilyas *et al.,* 2014 |
| DH (Cr x Ha, Cd x Ka), WW |  | + |  |  |  | Borràs-Gelonch et al., 2012 |
| 3 RIL pop (Weimai 8 x Luohan 2/Yannong 19/ Jimai 20), 4 env |  |  | + |  | Kernel length | Cui *et al.,* 2014 |
| DH (Excalibur x Kukri) WL | + |  |  |  | TCA organic acids, Glaucousness, grains per spike, harvest index | Hill *et al.,* 2013 |
| RIL (ND3331 X Tibetan semi-wild wheat acc. Zang 1817), WW |  | + |  |  | Spike length, grains per spike | Liu *et al.,* 2014 |
| RIL (Ventnor X Karl 92), WW & heat |  |  |  |  | Membrane damage & chlorophyll content | Talukder *et al.,* 2014 |
| DH (CD87/Katepwa),WW |  |  |  | + | Oil concentration | Moore *et al.,* 2015 |

**References for Table 6 (additional references in main text)**

Borràs-Gelonch, G., Rebetzke, G.J., Richards, R.A., and Romagosa, I. (2012). Genetic control of duration of pre-anthesis phases in wheat (*Triticum aestivum L.*) and relationships to leaf appearance, tillering, and dry matter accumulation. *J. Exp. Bot.* 63, 69- 89.

Cui, F., Zhao, C., Ding, A., Li, J., Wang, L., Li, X. et al (2014). Construction of an integrative linkage map and QTL mapping of grain yield-related traits using three related wheat RIL populations. *Theor. Appl.Genet.* 127, 659- 675.

Griffiths, S., Simmonds, J., Leverington, M., Wang, Y., Fish, L., Sayers, L. et al (2009). Meta-QTL analysis of the genetic control of ear emergence in elite European winter wheat germplasm. *Theor. Appl.Genet.* 119, 383- 395.

Huang, X., Cöster, H., Ganal, M., and Röder, M. (2003). Advanced backcross QTL analysis for the identification of quantitative trait loci alleles from wild relatives of wheat (*Triticum aestivum L*.). *Theor. Appl.Genet.*106, 1379- 1389.

Ilyas, M., Ilyas, N., Arshad, M., Kazi, A.G., Kazi, A.M., and Waheed, A. (2014). QTL mapping of wheat doubled haploids for chlorophyll content and chlorophyll fluorescence kinetics under drought stress imposed at anthesis stage. *Pak. J. Exp. Bot.* 46, 1889- 1897.

Kumar, N., Kulwal, P., Balyan, H., and Gupta, P. (2007). QTL mapping for yield and yield contributing traits in two mapping populations of bread wheat. *Mol. Breeding.* 19, 163- 177.

Liu, G., Jia, L., Lu, L., Qin, D., Zhang, J., Guan, P. et al (2014). Mapping QTLs of yield-related traits using RIL population derived from common wheat and Tibetan semi-wild wheat. *Theor. Appl.Genet.* 127, 2415- 2432.

Moore, C., Richards, R., and Rebetzke, G. (2015). Phenotypic variation and QTL analysis for oil content and protein concentration in bread wheat (*Triticum aestivum L.*). *Euphytica* 1- 12.

Morgan, J., and Tan, M. (1996). Chromosomal location of a wheat osmoregulation gene using RFLP analysis. Funct. Plant Biol. 23, 803- 806.

Peng, Z., Li, X., Yang, Z., and Liao, M. (2011). A new reduced height gene found in the tetraploid semi-dwarf wheat landrace Aiganfanmai. *Genet. Mol. Res.* 10, 2349- 2357.

Pinto, R.S., Reynolds, M.P., Mathews, K.L., Mcintyre, C.L., Olivares-Villegas, J.-J., and Chapman, S.C. (2010). Heat and drought adaptive QTL in a wheat population designed to minimize confounding agronomic effects. *Theor. Appl.Genet.* 121, 1001- 1021.

Rebetzke, G.J., Condon, A.G., Farquhar, G.D., Appels, R., and Richards, R.A. (2008). Quantitative trait loci for carbon isotope discrimination are repeatable across environments and wheat mapping populations. *Theor. Appl.Genet.* 118, 123-137.

Talukder, S.K., Babar, M.A., Vijayalakshmi, K., Poland, J., Prasad, P.V., Bowden, R. et al (2014). Mapping QTL for the traits associated with heat tolerance in wheat (*Triticum aestivum L.*). *BMC Genet.* 15, 97.

Yang, D.-L., Jing, R.-L., Chang, X.-P., and Li, W. (2007). Identification of quantitative trait loci and environmental interactions for accumulation and remobilization of water-soluble carbohydrates in wheat (*Triticum aestivum L*.) stems. *Genetics*. 176, 571- 584.
